# Mitochondrial remodeling in heart failure with reduced ejection fraction

**DOI:** 10.3389/fphys.2026.1958014

**Published:** 2026-10-07

**Authors:** Xin Chen

**Affiliations:** School of Life Science and Technology, Shandong Second Medical University, Weifang, China

**Keywords:** electron transport chain, heart failure, metabolic reprogramming, mitochondrial dynamics, mitochondrial membrane permeability transition, mitochondrial remodeling, mitophagy, oxidative stress

## Abstract

Extensive mitochondrial remodeling occurs during heart failure with reduced ejection fraction (HFrEF), yielding either protective adaptations that mitigate dysfunction or detrimental alterations that accelerate disease progression. Central to this remodeling are maladaptive metabolic reprogramming, featured by aberrant fuel preference and electron transport chain deficiency, which underlie the depletion of bioenergetic reserves. Concurrently, alterations in reactive oxygen species (ROS)-producing sites and antioxidant system elevate mitochondrial ROS production while diminishing ROS scavenging capacity, fostering a progressively deteriorating redox environment that perpetuates mitochondrial dysfunction. Additional maladaptive changes, including impaired mitochondrial biogenesis, fission-biased mitochondrial dynamics, mitophagy dysregulation, increased mitochondrial membrane permeability, and activation of pro-inflammatory responses, further disrupt mitochondrial homeostasis and exacerbate cardiac injury. Conversely, remodeling of the mitochondrial Ca^2+^ transport system provides adaptive restriction of mitochondrial Ca^2+^, conferring protection against apoptosis. In this review, I will summarize recent evidence demonstrating the molecular and pathophysiological mechanisms related to mitochondrial remodeling in HFrEF. I will delineate the signaling axes and factors that mediate distinct mitochondrial functions and specify how they are remodeled during the progression of heart failure. Different HFrEF etiologies are associated with distinct mitochondrial pathologies, and these will be comprehensively reviewed. I will also discuss the therapeutic potential of mitochondrial targeting in preclinical models and how these insights are translated into novel clinical strategies to improve heart failure management.

## 1 Introduction

Heart failure is a multifaceted clinical syndrome defined by the heart’s inability to maintain adequate cardiac output to meet systemic metabolic demands. Despite therapeutic and scientific advances, heart failure remains a leading cause of morbidity and mortality worldwide, with the prevalence continuing to rise as the population ages (Savarese et al., 2023). Heart failure is etiologically heterogeneous, with major contributors including ischemic injury from coronary artery disease and myocardial infarction (MI), chronic pressure overload from hypertension, and hemodynamic stress from valvular heart disease (Benjamin et al., 2019; Grossman et al., 1975). Heart failure represents a spectrum of ventricular dysfunction, ranging from predominant systolic failure (reduced contractility and ejection of blood) to diastolic failure (impaired relaxation and filling of blood), with many patients exhibiting a mixed phenotype affecting one or both ventricles (Bernardo et al., 2010; Bursi et al., 2006; De Keulenaer and Brutsaert, 2011). The pathogenesis of heart failure involves multiple interconnected mechanisms, including oxidative stress, metabolic derangement, inflammatory responses, neurohormonal dysregulation, and myocardial remodeling, all of which contribute to progressive cardiac deterioration (Tanai and Frantz, 2015). Approximately 50% of all heart failure cases are associated with impaired left ventricular systolic function and are classified as heart failure with reduced ejection fraction (HFrEF) (Cannata et al., 2026). HFrEF is defined by a left ventricular ejection fraction below 40% and is characterized by progressive left ventricular enlargement and adverse cardiac remodeling (Murphy et al., 2020). In clinical practice, coronary artery disease, MI, and hypertension are the most common underlying causes of HFrEF, and the etiology can be recapitulated by pharmacologic models or surgical models of ischemia- or pressure overload-induced heart failure (Bloom et al., 2017; Levy et al., 1996; McMurray et al., 2012). The pathophysiological heterogeneity of heart failure subtypes underscores the need for further research into the molecular basis of distinct heart failure subtypes, which is a prerequisite of developing effective therapeutic approaches.

Mitochondria occupy approximately one-third of the volume of adult cardiomyocytes and are the primary sites of cellular energy production. Under pathological stress, the inherent limitations of mitochondrial energy metabolism run counter to the escalating energy demand of the heart, creating a bioenergetic mismatch that is widely recognized as the primary mechanism linking mitochondrial dysfunction and HFrEF (Allard et al., 1994; Gorski et al., 2015). However, mitochondria are far more than the cell’s “powerhouse”. The view of mitochondria as mere ATP generators has given way to a broader understanding of these organelles as multifunctional regulators of cardiac metabolism, redox balance, calcium dynamics, inflammatory responses, and cell viability (Glover et al., 2024; Marchi et al., 2023; Pietrocola et al., 2015; Rizzuto et al., 2012; Spinelli and Haigis, 2018; Wong et al., 2017). Therefore, mechanisms that connect mitochondrial remodeling to the pathogenesis of HFrEF extend beyond the notion of bioenergetic failure. Furthermore, mitochondrial remodeling in HFrEF is a heterogeneous process that can not be uniformly categorized as maladaptive. On the contrary, it encompasses protective adaptations that mitigate contractile dysfunction and energy deficits. In this review, I will discuss both the adaptive and maladaptive mitochondrial alterations in HFrEF, elucidate the molecular and pathophysiological mechanisms underlying these remodeling processes, and clarify how they contribute to heart failure progression. I will also provide an overview of mitochondria as a promising preclinical target and discuss how these mechanistic insights are translated into innovative clinical approaches to improve heart failure outcomes. The aim of the review is to provide new insights into the molecular network governing mitochondrial remodeling in HFrEF and offer a theoretical foundation for studies on heart failure pathogenesis. Given the etiological heterogeneity of HFrEF, the mitochondrial remodeling events described herein may not be universally generalizable across all HFrEF models. Accordingly, each remodeling phenotype is presented alongside the specific model(s) in which it has been validated, and extrapolation to untested HFrEF models remains to be established. A comprehensive summary of mitochondrial alterations across different HFrEF etiologies and their associated molecular mechanisms is provided in the accompanying table (**Table 1**).

**Table 1:** Mitochondrial alterations in HFrEF and the associated molecular mechanisms.

Mitochondrial alterations	HFrEF models	Molecular mechanisms
FAO suppression	human HFrEF	Expression of MCAD, LCAD, and LCHAD is decreased because PPARα is downregulated
	human HFrEF, mouse PO	Mrpp2, a NAD^+^-dependent SDR that promotes retinol oxidation and enhances PPARα/RXRα signaling, is downregulated
	human ICM, human DCM, mouse PO	PPARα is activated by AMPK, which is downregulated and inactivated by PRL2
	rat ICM, mouse PO	CD36 and FABP, which facilitate fatty acid uptake and intracellular transport, are downregulated
Impaired FAO, ETC gene expression, and mitochondrial biogenesis caused by PGC-1α deficiency	mouse ICM, mouse PO	PGC-1α expression is activated by Pim kinases, which are downregulated in the indicated models
	human ICM, human DCM, mouse PO	PGC-1α expression is activated by KLF9, which is downregulated in the indicated models
	human ICM, human DCM, mouse PO, mouse ICM	PGC-1α expression is repressed by NOX4, which is upregulated in the indicated models
	human DCM, human ICM, mouse PO	PGC-1α expression is repressed by DYRK1B-STAT3, and DYRK1B is upregulated in the indicated models
	human DCM, mouse ICM	LARP7 acts through SIRT1 to maintain PGC-1α activity, and LARP7 is downregulated in the indicated models
	human ICM, human DCM, mouse PO, mouse I/R	S100a8/a9 suppresses PGC-1α expression via TLR4-ERK signaling, and S100a8/a9 is upregulated in the indicated models
	human DCM, mouse PO	PGC-1α expression is repressed by HEY2, which is upregulated in the indicated models
Enhanced FAO, ETC gene expression, and mitochondrial biogenesis caused by PGC-1α activation	human HFrEF, mouse PO	PGC-1α expression is activated by BRD4, which is upregulated in the indicated models
Upregulation of FAO	human DCM, mouse PO	MeCP2, which represses the expression of genes involved in FAO, is downregulated in the indicated models
Enhanced glycolysis and suppressed pyruvate oxidation	human HFrEF, mouse HFrEF	GLUT1 expression and PFK-1 activity are upregulated, but genes involved in pyruvate transport and oxidation, like MCT1, PDH, and MPCs, are downregulated
Glycolysis suppression	human ICM, human DCM, mouse I/R, mouse PO	The expression of Hexokinase II is downergulated
Glycolysis suppression	human ICM, human DCM, mouse PO	PKM1 expression is downregulated
	human DCM, human ICM, mouse PO	PKM1 activity is competitively inhibited by PKM2
Upregulation of ketone oxidation	human HFrEF, mouse HFrEF	Ketolytic enzymes, like BDH1, BDH2, and OXCT1, are upregulated
BCAA catabolism suppression	human DCM, human ICM, mouse PO	KLF15 is downregulated, contributing to reduced expression of BCAT2, BCKD, and BCKD phosphatase PP2Cm; GRSF1, which sustains BCKD expression by stabilizing its mRNA, is downregulated
ETC gene suppression at epigenetic level	human DCM, mouse ICM	METTL4 is upregulated, which increases 6mA abundance within mtDNA and disrupts transcription initiation complex assembly
ETC gene suppression at transcriptional level	mouse PO	PGC-1β expression is downregulated
	mouse ICM	DECR1 is downregulated, resulting in reduced expression of ETC subunits derived from complex I, II, and IV
	human ICM, mouse ICM, mouse PO	USP10, which prevents ubiquitin-dependent silencing of ETC genes, is downregulated
ETC gene suppression at post-transcriptional level	human HFrEF, mouse PO	Mrpp2, which mediates the processing of RNA precursors into mature RNA, is downregulated
ETC activity suppression	human ICM, human DCM, mouse I/R	ZIP13, which elevates mitochondrial iron levels to preserve ETC function, is downregulated
	human ICM, mouse PO	H_2_S serves as an electron donor, and 3-MST, which promotes H_2_S synthesis, is downregulated
	mouse I/R	Increased cTnI phosphorylation facilitates its binding to ATP synthase and promotes ATP hydrolysis
	human DCM, mouse PO	Cardiolipin maintains the activity of ETC complexes and the respiratory capacity. Enzymes that mediate cardiolipin biogenesis and remodeling, including CLS, tafazzin, and MLCLAT, are downregulated
Increased acetylation of mitochondrial proteins	human DCM, human ICM, mouse PO	SIRT3 expression is decreased and NAD^+^ levels are reduced
Increased S-nitrosylation of mitochondrial proteins	human DCM, mouse PO	GSNOR, which inhibits GSNO-mediated protein S-nitrosylation, is downregulated
	human DCM, rat ICM, mouse I/R, mouse PO	nNOS, which promotes S-nitrosylation of cytochrome *c* oxidase, is upregulated
Increased mitochondrial Ca^2+^ uptake through the MCU	mouse PO	uPAR, which increases IP_3_R-dependent SR Ca^2+^ release and enhances MCU-mediated Ca^2+^ uptake, is upregulated
	human nonICM, mouse PO	MCU expression is upregulated
Decreased mitochondrial Ca^2+^ uptake through the MCU	human HFrEF	MICU3 potentiates MCU activity and is downregulated
	human ICM, human non-ICM	MICU1 and MICU2, two inhibitory subunits of the MCU complex, are upregulated; the MICU1/MCU ratio is elevated
	mouse ICM	MCUB is incorporated into the MCU complex and displaces MCU from the complex
	human ICM, mouse ICM, mouse DCM	DWORF, which increases peak Ca^2+^ transient amplitutde and promotes MCU expression, is downregulated
	human ICM, mouse ICM, mouse PO	UCP2, which reduces mitochondrial membrane potential and MCU-mediated Ca^2+^ uptake, is upregulated
Decreased mitochondrial Ca^2+^ uptake through the VDAC1/Grp75/IP3R1/2 complex	human ICM, mouse PO	FMO2, which promotes the assembly of the VDAC1/Grp75/IP3R1/2 complex, is downregulated
	human DCM, mouse PO	MFN2, which stabilizes the VDAC1/Grp75/IP3R1/2 complex, is downregulated
	human ICM, mouse ICM	Mtus1A, which facilitates the assembly of the VDAC1/Grp75/IP3R1/2 complex, is downregulated
Increased mitochondrial Ca^2+^ efflux through NCE	human ICM, human DCM, animal PO	Cytosolic Na^+^ concentration is elevated, driving increased mitochondrial Na^+^ influx and Ca^2+^ efflux through NCE
Increased ROS production through the ETC	human HFrEF, mouse PO, mouse ICM	Hyperacetylation of ATP synthase inhibits its function and induces eletron transport defects
	human I/R, mouse I/R	Succinate accumulation and rapid oxidation generates ROS through reverse electron transport at complex I
Reduced ROS production through the ETC	mouse I/R	GPD2, which mediates ROS production, is downregulated by miR-210
	rat I/R	NDUFAF2, which promotes complex I assembly, is downregulated
Increased ROS production from non-ETC source	human ICM, human DCM, mouse PO, mouse ICM	NOX4 expression is upregulated
	human ICM, mouse PO	MAO expression is elevated
	pig I/R	p66Shc is activated and translocated to mitochondria
Increased antioxidant capacity	human DCM, human ICM	Pentose phosphate pathway is activated by β3AR signaling and NADPH production is elevated
Increased antioxidant capacity	human DCM	SOD2 and GPX1 are upregulated
Decreased antioxidant capacity	human HFrEF, mouse PO, mouse DCM	NAD^+^ is a precursor for NADPH. NMNAT, which mediates NAD^+^ synthesis, is downregulated
	human ICM, rat PO	Anaplerosis is enhanced and consumes NADPH
	human ICM, mouse ICM	NFE2L3, which suppresses the expression of SOD2, GPX4, and peroxiredoxin 2, is upregulated
	human ICM, mouse ICM	FOXO1/3 activates SOD2 and catalase expression. SIRT1, which sustains FOXO1/3 function, is downregulated
	human ICM, human DCM, mouse PO, mouse ICM	IDH2, which promotes NADPH production, is inactivated (human and mouse ICM); IDH2 is downregulated (human DCM and mouse PO)
	human DCM	Thioredoxin 2 is downregulated
Increased mPTP opening	human DCM, human ICM, mouse I/R, rat PO	Epac1, which stablizes the VDAC1/Grp75/IP3R1/2 complex and enhances SR-to-mitochondria Ca^2+^ transfer, is upregulated
	human DCM, mouse PO, mouse ICM	GRK2, which sensitizes mPTP to Ca^2+^, is upregulated
	human DCM, mouse PO	mecciRNAs, which maintain high TRAP1 and low CypD levels to inhibit mPTP opening, are degraded
	human DCM, human ICM, mouse PO	Increased CypD acetylation enhances its binding affinity to OSCP and induces mPTP opening
	rat I/R	The interactions within the CypD/VDAC1/Grp75/IP3R1/2 complex are enhanced, resulting in mitochondrial Ca^2+^ overload
Suppressed mPTP opening	human DCM, human ICM, mouse ICM	Increased HO-1 expression elevates CO production, which suppresses mPTP opening
	human HFrEF, mouse I/R	Pim-1 kinase is translocated to mitochondria and suppresses mPTP opening (mouse); Pim-1 expression is upregulated (human)
Suppressed mitochondrial fission	human DCM, mouse PO	FUNDC1, which activates FIS1 expression through CREB, is downregulated
Upregulated mitochondrial fission	human HFrEF, rat DCM, rat ICM	DRP-1 expression is upregulated
Impaired mitochondrial fusion	human DCM, human ICM	OPA1 is downregulated through post-translational modifications
Impaired mitochondrial fusion	rat ICM	MFN1 is phosphorylated and suppressed by βIIPKC
	human DCM, mouse PO	MFN2 is downregulated
Upregulated mitophagy	human hypoxia	DEPP1 activates mitophagic program. HIF, which potentiates DEPP1 expression, is upregulated
Suppressed mitophagy	mouse PO	Mitochondrial translocation of DRP-1 is suppressed
	human DCM, human ICM, mouse PO	AMPKα2 phosphorylates and activates PINK1. AMPKα2 expression is downregulated and its activity is suppressed by PRL2
	human DCM, mouse ICM, mouse PO	SPOP, which promotes the ubiquitination and degradation of TFEB, is upregulated
	human ICM, mouse ICM	ANXA2, which induces the proteasomal degradation of PHB2, is upregulated
	mouse I/R	Lysine crotonylated IDH3a suppresses BNIP3-mediated mitophagy and IDH3a Kcr levels are increased
	human ICM, rat ICM	miR-130a, which suppresses FUNDC1-mediated mitophagy, is upregulated
Potentiated inflammatory responses mediated by mitochondria	human ICM, mouse ICM, mouse PO	USP10 deubiquitinates MTX2 to preserve cristae integrity, prevent mtDNA release, and inhibit the activation of RIPK3-NF-ĸB pathway; USP10 is downregulated
	human DCM	STMP1, which maintains cristae integrity through interactions with the components of the cristae organizing complexes, is downregulated, resulting in mtDNA release
Suppressed inflammatory responses mediated by mitochondria	human ICM, mouse ICM, mouse PO	ZBP1, which inhibits RIPK3-NF-ĸB pathway activity and inflammatory cytokines expression, is upregulated
Alternative splicing dysregulation	human DCM, mouse PO	DCRT, which inhibits third exon skipping of NDUFS2, is downregulated
	rat hypoxia	A spliced variant of BNIP3 interferes with BNIP3’s function

DCM indicates dilated cardiomyopathy-induced heart failure; hypoxia, hypoxia-induced stress; ICM, ischemic cardiomyopathy-induced heart failure; I/R, ischemia/reperfusion-induced heart failure; PO, pressure overload-induced heart failure; HFrEF indicates a heart failure model for which the etiology has not been identified.

## 2 Fuel preference in the failing heart

HFrEF is fundamentally a disease of energy starvation, driven largely by diminished mitochondrial oxidative capacity. This metabolic deficit stems from a pathological shift in substrate utilization: fatty acid and glucose oxidation are suppressed, while glycolysis and ketone oxidation are paradoxically upregulated (Bertero and Maack, 2018; Lopaschuk et al., 2021). The following section synthesizes recent findings on the remodeling of metabolic enzymes and the transcriptional networks that govern oxidative metabolism in HFrEF and specifies how these remodeling events precipitate the energetic failure.

### 2.1 Fatty acid metabolism in heart failure

The transcriptional program governing fatty acid uptake, mitochondrial import, and β-oxidation is directed by estrogen-related receptor α (ERRα), peroxisome proliferator-activated receptor α (PPARα), and peroxisome proliferator-activated receptor gamma coactivator-1α (PGC-1α), with synergistic activation being reserved for a subset of the target genes (Campbell et al., 2002; Huss et al., 2004, 2002; Montaigne et al., 2021; Vega et al., 2000; Watanabe et al., 2000). All three regulators are subject to control by mammalian target of rapamycin complex 1 (mTORC1) under basal conditions (Huss et al., 2004; Shende et al., 2011). Despite differential regulation of metabolic gene expression, both human nondiabetic failing hearts (with dilated cardiomyopathy (DCM) or ischemic cardiomyopathy (ICM)) and the pressure overload (PO)-induced mouse heart failure model exhibit reduced reliance on fatty acid oxidation (FAO) for oxidative metabolism (Aubert et al., 2016; Barger and Kelly, 1999; Bedi et al., 2016). In the hearts of patients with HFrEF, attenuation of FAO is associated with decreased expression of medium-chain acyl-CoA dehydrogenase (MCAD), long-chain acyl-CoA dehydrogenase (LCAD), and long-chain 3-hydroxyacyl-CoA dehydrogenase (LCHAD), which catalyze the first and third steps of the FAO cycle (Sack et al., 1996). PPARα protein levels decrease markedly in the human failing hearts, which contributes to the diminished expression of a variety of FAO enzymes that include MCAD and LCAD (Karbowska et al., 2003). The downregulation of FAO is also attributable to the reduced expression of mitochondrial ribonuclease P protein 2 (Mrpp2), a NAD(P)H-dependent short-chain dehydrogenase/reductase (SDR) that has been implicated in retinol oxidation and PPARα/retinoid X receptor α (RXRα) signaling (Barger et al., 2000; Bedi et al., 2016; Kelly, 2003; Luo et al., 2014; van Vlies et al., 2007; Walker et al., 2023; Yang et al., 2021). Additional FAO control arises from AMP-activated protein kinase (AMPK)-dependent PPARα activation (Jin et al., 2022; Zhang et al., 2021). In the human (with DCM or ICM) and mouse (PO-induced) heart failure models, both the expression and activity of AMPK are downregulated, leading to suppressed FAO (Han et al., 2025; Wang et al., 2018). PGC-1α coactivates PPARα and ERRα to induce FAO gene expression and is subject to positive transcriptional control by Pim kinase (Pim-1, Pim-2, and Pim-3) and Krüppel-like factor 9 (KLF9) (**Figure 1**) (Din et al., 2014; Zhang et al., 2024). The Pim kinase family consists of constitutively active serine/threonine kinases that are highly expressed in the postnatal heart and are implicated in promoting cell proliferation and survival (Din et al., 2014). KLF9, a member of the Krüppel-like family of zinc finger domain transcription factors, maintains mitochondrial homeostasis by integrating cardiac energy metabolism, mitochondrial dynamics, and mitophagy (Zhang et al., 2024). The suppression of the Pim kinase and KLF9 signaling axes in the ischemic or PO-induced heart failure models abrogates their positive regulation of FAO, thereby impairing fatty acid utilization and energy metabolism (**Figure 1**) (Din et al., 2014; Zhang et al., 2024). Notably, Pim-1 protein levels are elevated in the human failing hearts, possibly serving as a compensatory mechanism to enhance fatty acid utilization (Muraski et al., 2007). Decreased expression of proteins responsible for fatty acid uptake and intracellular trafficking also contributes to the impaired fatty acid metabolism in animal models of heart failure. The expression of cluster of differentiation 36 (CD36) and fatty acid binding protein (FABP) is decreased in the infarcted rat hearts and the PO-induced mouse failing hearts (Heather et al., 2006; Sung et al., 2017). However, in human heart failure, these two transporters exhibit distinct dynamics: both remain unchanged in ischemic heart failure but are upregulated in the DCM-affected failing hearts (García-Rúa et al., 2012). This disparity may be attributable to differences between animal and human hearts in their capacity to compensate for the decline in fatty acid metabolism. Fatty acids are the heart’s dominant energy substrate, contributing approximately 40-60% of mitochondrial ATP production under normal conditions (Lopaschuk et al., 2021). Therefore, downregulation of FAO through the maladaptive mechanisms described above deprives the heart of its most efficient source of ATP. In an apparent attempt to bolster FAO and ATP synthesis efficiency, the human (with DCM) and mouse (PO-induced) failing hearts relieve methyl-CpG-binding protein 2 (MeCP2)-mediated repression of fatty acid metabolism and induces carnitine palmitoyl transferase 1a (CPT1a) expression to facilitate the entry of long-chain fatty acid (LCFA) into mitochondria (Carley et al., 2026; Kim et al., 2020; Mayer et al., 2015; Padmanabhan et al., 2020). However, acutely increasing CPT1a reduces the contribution of LCFA to oxidation to the tricarboxylic acid (TCA) cycle, suggesting that CPT1a induction is a maladaptive response to elevated metabolic stress (Carley et al., 2026; Lewandowski et al., 2013).

**Figure 1:**
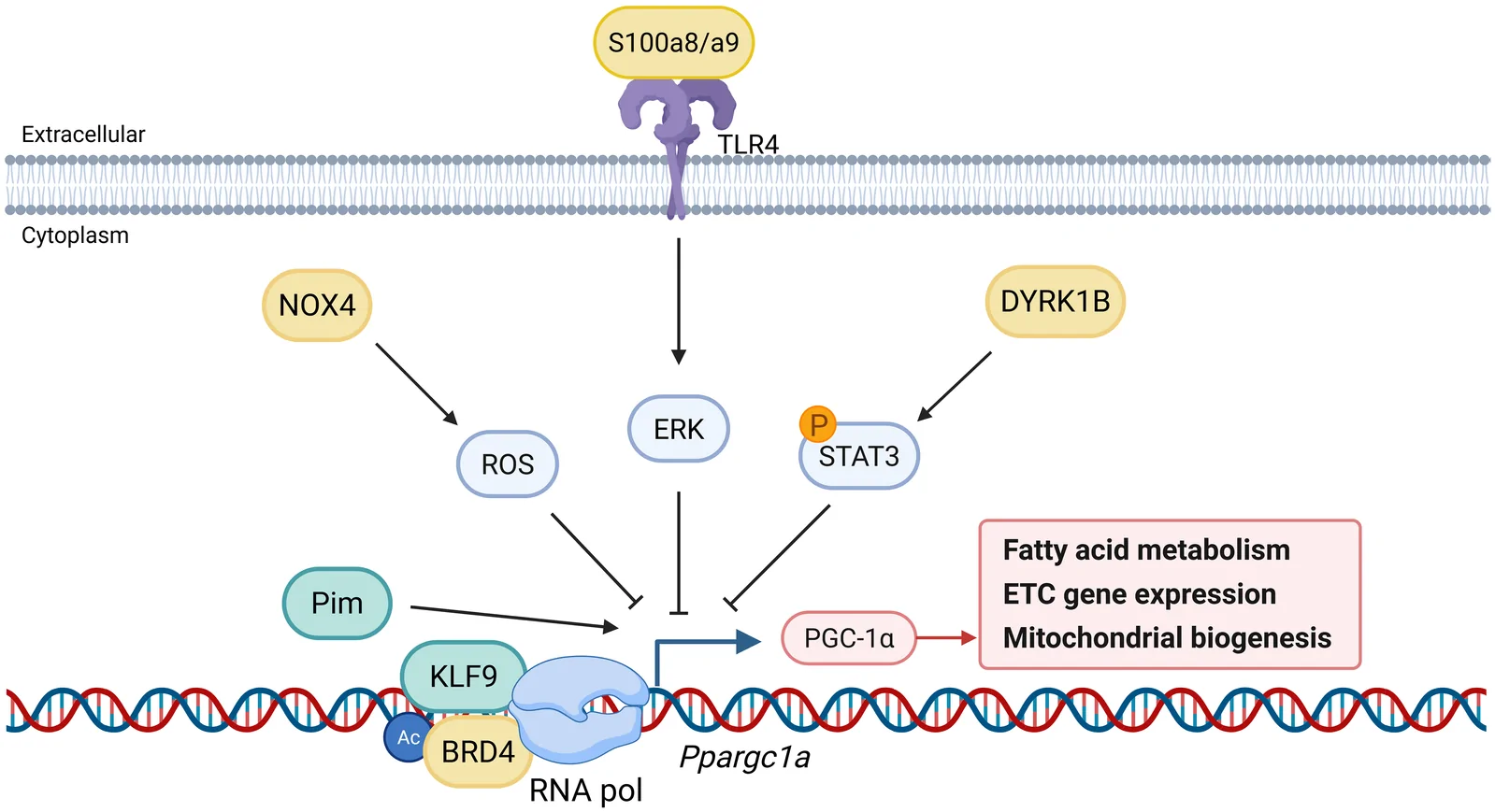
Regulation of PGC-1α (*Ppargc1a*) expression in heart failure with reduced ejection fraction. Bromodomain-containing protein 4 (BRD4) binds to acetyl-lysine (Ac) in histone tails and recruits transcriptional machinery (RNA polymerase, RNA pol) to the promoter of *Ppargc1a* to initiate its transcription. In pressure overload (PO)-induced mouse failing hearts and in human HFrEF, increased BRD4 expression upregulates *Ppargc1a* transcription. Krüppel-like factor 9 (KLF9) directly binds to the promoter of *Ppargc1a* to promote its transcription. The downregulation of KLF9 in the human (with dilated cardiomyopathy (DCM) or ischemic cardiomyopathy (ICM)) and mouse (PO-induced) failing hearts suppresses *Ppargc1a* expression. Pim kinases (Pim-1, Pim-2, and Pim-3) positively regulate *Ppargc1a* transcription, and their downregulation in the ischemic or PO-induced mouse failing hearts leads to reduced PGC-1α expression. Moreover, NADPH oxidase 4 (NOX4)-derived ROS suppresses *Ppargc1a* transcription. In the human (with ICM or DCM) and mouse (with ICM or PO-induced) failing hearts, elevated NOX4 expression leads to attenuated *Ppargc1a* expression. The inflammatory molecule S100a8/a9 activates Toll-like receptor 4 (TLR4), which transduces the signal via extracellular signal-regulated kinase (ERK) to suppress *Ppargc1a* transcription. S100a8/a9 expression is elevated in the human (with ICM or DCM) and mouse (PO- or I/R-induced) failing hearts, resulting in diminished PGC-1α expression. Dual-specificity tyrosine-regulated kinase 1B (DYRK1B) phosphorylates (P) signal transducer and activator of transcription 3 (STAT3) and facilitates its nuclear translocation, and phosphorylated STAT3 negatively regulates *Ppargc1a* transcription. In the human (with ICM or DCM) and mouse (PO-induced) failing hearts, DYRK1B expression is upregulated, leading to decreased PGC-1α expression. Given PGC-1α’s established role in promoting fatty acid metabolism, electron transport chain (ETC) gene expression, and mitochondrial biogenesis, the signaling axes/factors mentioned above serve as potent modulators of these mitochondrial functions. The proteins colored in green are the ones whose expression is downregulated in a particular heart failure model, and the proteins colored in yellow are the ones whose expression is upregulated in a particular heart failure model. Created in BioRender. Chen, X. (2026) https://BioRender.com/oq4hdcx.

Conversely, metabolic remodeling in the model of heart failure with preserved ejection fraction (HFpEF) is characterized by increased FAO dependence for bioenergetics, accompanied by coordinated upregulation of proteins controlling fatty acid uptake, trafficking, and oxidative catabolism (Koay et al., 2025; Yoshii et al., 2024). However, the enhanced FAO dependence in HFpEF mice does not translate into greater ATP-producing capacity. Rather, net FAO-derived ATP generation is compromised, potentially due to diminished CPT1b expression and hyperacetylation-mediated functional loss of very long-chain acyl-CoA dehydrogenase (VLCAD) and trifunctional enzyme subunit α (HADHA) (Koay et al., 2025; Tong et al., 2021; Yoshii et al., 2024).

### 2.2 Glucose metabolism in heart failure

In the model of heart failure with reduced ejection fraction (HFrEF), cardiac metabolism is reprogrammed toward increased reliance on glucose, leading to relatively enhanced glycolysis that is uncoupled from oxidation (Allard et al., 1994; Diakos et al., 2016; Lei et al., 2004; Pound et al., 2009; Zhabyeyev et al., 2013; Zhang et al., 2013). Mechanistically, the failing hearts upregulate glucose transporter 1 (GLUT1) expression and phosphofructokinase 1 (PFK-1) activity to increase glycolytic flux, but simultaneously represses genes required for pyruvate mitochondrial import and oxidation, a pattern conserved in both the human and mouse models (Diakos et al., 2016; Fernandez-Caggiano et al., 2020; Heggermont et al., 2017; Lee et al., 2020; Lei et al., 2004). Metabolomic profiling of myocardial biopsies from patients with HFrEF demonstrates a marked accumulation of pyruvate and other glycolytic intermediates, indicating suppressed pyruvate oxidation (Badolia et al., 2020; Cluntun et al., 2021). Since fatty acids are the least oxygen-efficient substrate for ATP production, the metabolic shift away from fatty acids and toward glucose has been interpreted as adaptive attempt to improve oxygen efficiency. However, this compensatory effort is insufficient to completely resolve the cardiac energy deficit since the capacity for glycolysis-derived ATP production is inherently limited (Allard et al., 1994). Glycolytic ATP production is further constrained by the deficiency of key glycolytic enzymes in heart disease models. Hexokinase II expression is diminished in the mouse hearts in response to ischemia/reperfusion (I/R), in the PO-induced mouse failing hearts, and in the hearts of patient with ICM or DCM (Dong et al., 2015; Ko et al., 2026; Wu et al., 2011). Pyruvate kinase muscle isozyme 1 (PKM1) is also downregulated, and its activity is competitively inhibited by PKM2 in both the human (with DCM or ICM) and mouse (PO-induced) failing hearts, collectively limiting the cellular response to glycolysis-inducing signals (Li et al., 2021; Tang et al., 2023). Therefore, while the upregulation of selective glycolytic enzymes improves energy metabolism and proves adaptive, other glucose metabolism-related alterations are considered maladaptive since they contribute to myocardial “energy starvation”.

Glycolysis is suppressed in the HFpEF model, as evidenced by reduced abundance of GLUT4 and hexokinase I and II (Koay et al., 2025). Glycolysis is similarly suppressed in the DCM-affected human failing hearts, owing to reduced expression of nearly all glycolytic enzymes (Flam et al., 2022). In these DCM failing hearts, acetyl-CoA levels are preserved, whereas the vast majority of TCA cycle intermediates are profoundly depleted, indicative of anaplerotic insufficiency or increased cataplerosis (Flam et al., 2022).

### 2.3 Ketone oxidation in heart failure

The expression of key ketolytic enzymes, including β-hydroxybutyrate dehydrogenase 1 and 2 (BDH1 and BDH2) and 3-oxoacid-CoA-transferase (OXCT1), are elevated in human and mouse models of HFrEF, increasing the contribution of ketone bodies to overall oxidative metabolism (Aubert et al., 2016; Bedi et al., 2016; Yoshii et al., 2024). Converging evidence from plasma measurements, transporter expression, and metabolite profiles indicates that ketone body delivery, uptake, and oxidation are all elevated in these murine failing hearts, constituting an adaptive response to intensified energetic stress (Aubert et al., 2016; Bedi et al., 2016). In the murine HFpEF model, ketone body transporters and oxidation enzymes show reduced abundance, reflecting downregulated ketone body utilization (Koay et al., 2025).

### 2.4 Branched-chain amino acid catabolism in heart failure

BCAA catabolism is suppressed in the failing myocardium. Branched chain amino acid transaminase 2 (BCAT2), branched-chain alpha-ketoacid dehydrogenase complex (BCKD), and BCKD phosphatase PP2Cm are key BCAA catabolic enzymes and their expression is decreased in the human failing hearts (with DCM or ICM) and the PO-induced mouse failing hearts because of the downregulation of the upstream regulator, Krüppel-like factor 15 (KLF15) (Haldar et al., 2010; Prosdocimo et al., 2014; Sun et al., 2016). Guanine-rich RNA sequence binding factor 1 (GRSF1), which enhances BCKD expression by stabilizing its mRNA, is downregulated in the human failing hearts (with DCM or ICM) and the PO-induced mouse heart failure model, resulting in diminished BCKD levels (Wang et al., 2026). Impaired BCAA catabolism leads to accumulation of BCAAs and BCAA catabolic mediators (e.g., branched-chain α-keto acids (BCKAs)) in the human (with DCM) and mouse (PO-induced) failing hearts, which compromises complex I-mediated respiration, fuels mitochondrial ROS production, and increases susceptibility to heart failure (McGarrah and White, 2023; Sansbury et al., 2014; Sun et al., 2016; Wang et al., 2026; White and Newgard, 2019). Therefore, the downregulation of BCAA catabolism in the failing hearts is considered maladaptive.

## 3 Mitochondrial electron transport chain deficiency in HFrEF

Through the oxidation of NADH and FADH_2_, the ETC establishes a proton-motive force essential for ATP generation. Impairment of ETC function consequently impairs mitochondrial bioenergetics and compromises cell viability. Clinically, mutations in both nuclear and mitochondrial genes encoding ETC subunits are established contributors to cardiac dysfunction and failure (Casademont and Miró, 2002). Nevertheless, the behavior of ETC complexes in HFrEF, in terms of both functional activity and expression remodeling, has not been comprehensively reviewed. I will therefore address this gap by presenting the functional derangements of ETC complexes in HFrEF and examining their regulation via epigenetic modifications, transcriptional control, and post-transcriptional mechanisms.

### 3.1 Alterations in ETC gene expression in HFrEF

In HFrEF, ETC gene expression is modulated at the epigenetic, transcriptional, and post-transcriptional levels, affecting both nuclear- and mitochondrial-encoded ETC subunits. Cardiomyocytes derived from both ischemic and nonischemic mouse failing hearts and the human (with DCM) failing hearts exhibit a pronounced increase in N^6^-methyladenine (6mA) abundance within mitochondrial DNA (mtDNA) (Zhang et al., 2024). This 6mA modification is catalyzed by methyltransferase-like protein 4 (METTL4), a pathogenic methyltransferase that is directly upregulated by p53 in response to heart failure-provoking stress (Zhang et al., 2024). Increased 6mA methylation at the promoters of mtDNA interferes with the assembly of the transcription initiation complex and therefore suppresses the expression of the mtDNA-encoded ETC genes, culminating in mitochondrial morphological and functional abnormalities (Zhang et al., 2024). Therefore, METTL4 upregulation in the indicated heart failure models is maladaptive.

A substantial fraction of nuclear- and mitochondrial-encoded ETC subunits are transcriptionally regulated by PGC-1α (Lehman et al., 2000; Rodgers et al., 2005). However, in the diseased myocardium, the transcriptional regulatory network governing oxidative phosphorylation operates through both PGC-1α-dependent and PGC-1α-independent mechanisms. Bromodomain and Extra-Terminal (BET) epigenetic reader proteins bind acetyl-lysine in histone tails and facilitate the recruitment and modulation of the transcriptional machinery at acetylated loci (Wu and Chiang, 2007). Bromodomain-containing protein 4 (BRD4), a member of the BET family, functions as a critical epigenetic reader and transcriptional regulator in the heart. In the human failing hearts and a mouse model of PO-induced heart failure, BRD4 potentiates the expression of nuclear-encoded ETC subunits either directly through synergy with ERRα or indirectly in a PGC-1α-dependent manner, contributing positively to cardiac energetic adaptation (**Figure 1**) (Arany et al., 2005; Kim et al., 2020; Li et al., 2025; Padmanabhan et al., 2020; Spiltoir et al., 2013). However, the majority of the remodeling events affecting ETC gene transcription are maladaptive, compromising oxidative phosphorylation and precipitating the energetic deficits observed in heart failure models. First of all, KLF9 deficiency reduces PGC-1α expression in both the human (DCM or ICM) and mouse (PO-induced) failing hearts (**Figure 1**) (Zhang et al., 2024). In parallel, dual-specificity tyrosine-regulated kinase 1B (DYRK1B) and signal transducer and activator of transcription 3 (STAT3) act synergistically to suppress PGC-1α and ETC gene expression in the human (with DCM or ICM) and mouse (PO-induced) failing hearts, while cardiac-specific overexpression of DYRK1B directly induces heart failure, indicative of the pathogenic role of the DYRK1B-STAT3 axis (**Figure 1**) (Zhuang et al., 2022). HEY2, a Hairy/Enhancer-of-split-related transcriptional repressor, colocalizes with histone deacetylase 1 (HDAC1) at the PGC-1α promoter to facilitate transcriptional repression. In the human (with DCM) and mouse (PO-induced) heart failure models, the upregulation of HEY2 contributes to decreased expression of PGC-1α and ETC genes (She et al., 2025; Wang et al., 2025). Moreover, CaMKIIδ relays the Gαq-dependent signals to repress ETC gene expression, selectively impairing complex I-linked respiration (Westenbrink et al., 2015). PGC-1β serves as an additional transcriptional regulator of ETC genes and its decline during chronic PO contributes to oxidative phosphorylation deficiency (Riehle et al., 2011). Interestingly, the mRNA levels of PGC-1β remain unchanged in the human failing hearts, suggesting that mechanisms governing PGC-1β expression diverge between human and animal models (Karamanlidis et al., 2010).

2,4-dienoyl-CoA reductase 1 (DECR1) is a short-chain dehydrogenase/reductase that controls β-oxidation and metabolism of unsaturated fatty acids. The downregulation of mitochondrial DECR1 in infarcted mouse hearts exerts a broad effect on the respiratory chain, reducing expression of subunits from mitochondrial complex I, II, and IV, and contributing to mitochondrial dysfunction (Duan et al., 2024). Notably, DECR1 is upregulated in the DCM-affected human failing hearts, possibly serving as a compensatory mechanism to potentiate ETC gene expression (Song et al., 2023). Ubiquitin-specific peptidase 10 (USP10) is a deubiquitinating enzyme that plays a protective role in the heart by stabilizing key mitochondrial proteins, including NADH dehydrogenase ubiquinone iron-sulfur protein 1 (NDUFS1) (Fu et al., 2026). In the human (with ICM) and mouse (with ICM or PO-induced) failing hearts, reduced USP10 expression causes extensive ETC gene downregulation, implicating a ubiquitin-dependent silencing mechanism (Li et al., 2025; Zhao et al., 2026). La ribonucleoprotein domain family member 7 (LARP7) functions as a cardioprotective gatekeeper that maintains mitochondrial homeostasis and cardiac function by stabilizing sirtuin 1 (SIRT1), a NAD^+^-dependent deacetylase. The downregulation of LARP7 leads to PGC-1α hyperacetylation, impairing its transcriptional activity and suppressing ETC gene expression in the human (DCM) and ischemic mouse failing hearts (Yu et al., 2021). Furthermore, S100a8/a9 proteins function primarily as damage-associated molecular patterns (DAMPs) or alarmins that drive inflammation and cardiac injury. They activate nuclear factor-kappa B (NF-κB) signaling and increase pro-inflammatory cytokines expression following I/R injury (Volz et al., 2012). Besides these pro-inflammatory roles, S100a8/a9 also participate in ETC gene regulation. By repressing PGC-1α, S100a8/a9 selectively target the transcription of NDUFS genes, impairing ETC complex I activity and inducing respiration defects in the mouse hearts in response to I/R injury (**Figure 1**) (Li et al., 2019). S100a8/a9 protein levels are also elevated in the hearts of patients with acute MI or DCM and in the PO-induced mouse failing hearts, contributing to energetic deficits (Ma et al., 2024; Yu et al., 2024b; Yuan et al., 2024). The inhibitory effect of S100a8/a9 on PGC-1α expression is possibly mediated through NOX4, a NADPH oxidase that is induced by S100a8/a9 in response to ischemic stress and exerts negative impact on PGC-1α expression (Ago et al., 2010; Vlad et al., 2025). These ETC gene-related mitochondrial alterations are also considered maladaptive as they exacerbate energetic failure in the indicated HFrEF models. The G protein-coupled receptor (GPCR) Latrophilin-2 (Lphn2) acts through p38 mitogen activated protein kinase (MAPK) to stimulate PGC-1α expression (Canovas and Nebreda, 2021; Lee et al., 2021; Puigserver et al., 2001). Cardiac Lphn2 deficiency predisposes to DCM, but whether DCM commonly involves the downregulation of Lphn2 remains to be determined (Kang et al., 2024). A meta-analysis of animal and human heart failure microarray data shows that the expression levels of ETC genes are universally downregulated in animal HFrEF models, whereas the ETC gene profile for human HFrEF is heterogeneous (Barth et al., 2011). The heterogeneity of human microarray data can be attributed to differences in sample preparation and medication use, which have a substantial impact on ETC gene expression.

At the post-transcriptional level, disruption of the RNA processing machinery further compromises ETC gene expression and causes respiratory dysfunction. mtDNA is transcribed as long polycistronic transcripts and processing of the RNA precursors by mitochondrial ribonuclease P (RNase P) and RNase Z endonucleases is a necessity for RNA maturation (Bhatta et al., 2021; D’Souza and Minczuk, 2018; Holzmann et al., 2008; Kotrys and Szczesny, 2019; Rackham et al., 2016). The downregulation of Mrpp2 in the human and the PO-induced mouse failing hearts causes accumulation of mitochondrial pre-RNA fragments, a defect that reduces the expression of mtDNA-encoded ETC subunits and impairs oxidative phosphorylation (Walker et al., 2023). Therefore, this post-transcriptional remodeling event caused by Mrpp2 deficiency is also regarded as maladaptive.

### 3.2 Modulation of the mitochondrial ETC activity in HFrEF

Multiple ETC complex subunits, including NDUFS1 of complex I, succinate dehydrogenase subunit B (SDHB) of complex II, and Ubiquinol-cytochrome *c* reductase, Rieske iron-sulfur polypeptide 1 (UQCRFS1) of complex III, are Fe-S containing proteins and are therefore susceptible to mitochondrial iron level fluctuations (Li et al., 2025). SLC39A13 (ZIP13) is a member of the zinc/iron-regulated transporter-like protein and functions to elevate mitochondrial iron content so as to preserve respiratory function (Li et al., 2025). In the mouse hearts, ZIP13 deficiency in response to I/R injury causes reduction in ETC complex activity and impairs mitochondrial respiration (Wang et al., 2021). ZIP13 expression is also significantly downregulated in the hearts of patients with ICM or DCM, pointing to a common defect in maintaining mitochondrial iron homeostasis (Li et al., 2025). Mitochondria are also responsive to hydrogen sulfide (H_2_S), which promotes mitochondrial biogenesis and serves as an electron donor to enhance oxidative phosphorylation (Elrod et al., 2007; Li et al., 2022; Módis et al., 2013). However, 3-mercaptopyruvate sulfurtransferase (3-MST), the enzyme that catalyzes H_2_S synthesis, is downregulated in the human ischemic failing hearts and a mouse model of PO-induced heart failure, reducing mitochondrial H_2_S availability and compromising cardiac energetics (Li et al., 2022). Additionally, emerging evidence indicates that sarcomeric proteins, while primarily responsible for cardiomyocyte contraction, also participate in the regulation of respiratory chain activity. Around 10% of the cardiac troponin I (cTnI) have intramitochondrial localization, and cTnI can bind to mitochondrial ATP synthase and enhance its ATP hydrolytic activity, thereby inhibiting ATP synthesis (Elezaby et al., 2024). In response to I/R injury, increased cTnI phosphorylation in the mouse hearts enhances its binding affinity to ATP synthase and markedly stimulates ATP hydrolysis, resulting in energetic deficits (Elezaby et al., 2024). Furthermore, impaired cardiolipin biogenesis and remodeling also contribute to mitochondrial respiration dysfunction. Cardiolipin is a unique phospholipid located in the inner mitochondrial membrane, where it stabilizes the ETC complexes (especially for cytochrome oxidase) and supports optimal respiration (Sparagna et al., 2007). In the human (DCM-affected) and mouse (PO-induced) failing hearts, enzymes that catalyze cardiolipin biosynthesis and remodeling, including cardiolipin synthase (CLS), tafazzin, and monolysocardiolipin acyltransferase (MLCLAT), are downregulated, resulting in cardiolipin deficiency and disruption of ETC function (Chatfield et al., 2014; Liu et al., 2024). Since alterations described above compromise ETC complex activity and lead to the decline of respirational functions, they are considered maladaptive.

## 4 Post-translational protein modifications that alter mitochondrial bioenergetics

### 4.1 Acetylation

Protein acetylation is the addition of an acetyl group to the ϵ-amino group on lysine residues of target proteins. Cellular acetylome is regulated by sirtuins, a group of NAD^+^-dependent deacetylases, and SIRT3 is the isoform that predominantly functions in mitochondria (Finkel et al., 2009; Imai et al., 2000; Landry et al., 2000; Onyango et al., 2002; Schwer et al., 2002). The relative abundance of mitochondrial lysine acetylation is increased in the human (with DCM or ICM) and mouse (PO-induced) failing hearts due to reduced SIRT3 expression and diminished NAD^+^ levels arising from mitochondrial complex I deficiency (Lee et al., 2016; Zhang et al., 2018). Protein hyperacetylation has been associated with impaired function for pyruvate dehydrogenase (PDH) complex, ATP synthase, and malate aspartate shuttle (MAS) proteins, contributing to a decline in oxidative phosphorylation efficiency (Lee et al., 2016; Zhang et al., 2018). Therefore, elevation of protein acetylation levels in heart failure is normally regarded as maladaptive. However, the assumption of a causal relationship between hyperacetylation and compromised bioenergetics has been challenged by a recent study showing a subtly altered mitochondrial respiratory capacity in hearts with highly acetylated mitochondrial proteome (Davidson et al., 2020). The study utilized a SIRT3/CrAT (carnitine acetyltransferase) dual knockout model, in which the population of acetylated peptides overlaps substantially with that of a heart failure model. Although the bioenergetic discrepancy between the two models could still be explained by the difference in the stoichiometry of acetylation, the study casts doubt on the idea that modest protein acetylation has a substantial impact on mitochondrial respiratory function.

### 4.2 S-Nitrosylation

S-nitrosylation is a redox-based post-translational modification that involves the covalent bonding of a nitric oxide group to specific cysteine residues within proteins. S-nitrosoglutathione reductase (GSNOR) catabolizes S-nitrosoglutathione (GSNO) and inhibits GSNO-mediated protein S-nitrosylation (Benhar et al., 2009; Liu et al., 2004). The downregulation of GSNOR in the human (with DCM) and mouse (PO-induced) failing hearts enhances S-nitrosylation and inactivation of adenine nucleotide translocase 1 (ANT1), thereby disrupting mitochondrial energy transport and culminating in respiratory defects (Sharer et al., 2002; Tang et al., 2023; Wang et al., 2009). Additionally, the levels of mitochondria-localized neuronal nitric oxide synthase (nNOS) are increased in the mouse hearts following I/R injury, and its expression is prominently elevated in the human (with DCM), mouse (PO-induced), and rat (with ICM) failing hearts, promoting S-nitrosylation of cytochrome *c* oxidase and resulting in mitochondrial respiratory dysfunction (Burkard et al., 2010; Damy et al., 2004, 2003). Therefore, elevated S-nitrosylation of mitochondrial proteins induced by alterations described above is maladaptive.

### 4.3 *O*-GlcNAcylation

*O*-linked β-N-acetylglucosamine (*O*-GlcNAc) cycling is mediated by the opposing actions of *O*-GlcNAc transferase (OGT), which adds GlcNAc to proteins, and *O*-GlcNAcase (OGA), which removes GlcNAc from proteins (Watson et al., 2010). While reducing myocardial *O*-GlcNAcylation by OGT deletion increases the heart’s susceptibility to MI-induced injury, inducing excessive *O*-GlcNAcylation through OGA inhibitors exacerbates cardiac dysfunction in post-MI mouse failing hearts (Lunde et al., 2012; Watson et al., 2010). Reflecting the need for balanced *O*-GlcNAcylation, acute OGT expression elevates mitochondrial *O*-GlcNAcylation and impairs respiratory chain function, whereas modest OGA inhibition enhances mitochondrial oxygen consumption and ATP synthesis (Banerjee et al., 2015; Hu et al., 2009; Ma et al., 2015; Umapathi et al., 2021).

## 5 Mitochondrial Ca^2+^ dynamics in HFrEF

Beyond its canonical role as a second messenger in myriad signaling pathways, Ca^2+^ operates at the mitochondrial level to fine-tune energy production in accordance with cellular demand. The machinery mediating mitochondrial Ca^2+^ influx and efflux has been rigorously delineated (Johnson et al., 2025; O’Rourke et al., 2021). Building on this foundation, the present section extends current understanding by examining how the mitochondrial Ca^2+^ transport apparatus is remodeled, functionally amplified or attenuated, during HFrEF progression, and how these alterations perturb Ca^2+^ handling in the failing heart.

### 5.1 Mitochondrial Ca^2+^ uptake through mitochondrial Ca^2+^ uniporter

MCU controls mitochondrial Ca^2+^ uptake and it functions in a complex consisting of MCU, essential MCU regulator (EMRE), the scaffold protein MCU regulator 1, the MCU paralog, and a family of regulatory proteins, including mitochondrial Ca^2+^ uptake 1 (MICU1), MICU2, and MICU3 (**Figure 2**) (Baughman et al., 2011; Csordás et al., 2013; D et al., 2023; De Stefani et al., 2011; Mallilankaraman et al., 2012b; Patron et al., 2014; Perocchi et al., 2010; Raffaello et al., 2013; Sancak et al., 2013). Both MICU1 and MICU3 exert a stimulatory effect on MCU (MICU1 inhibits MCU activity at low cytosolic Ca^2+^ concentrations), whereas MICU2 inhibits its channel opening activity (Csordás et al., 2013; Mallilankaraman et al., 2012b; Patron et al., 2014; Roman et al., 2024). The sigmoidal relationship between MCU-mediated Ca^2+^ uptake and cytosolic Ca^2+^ concentration allows for prompt Ca^2+^ uptake during peak Ca^2+^ transient and termination of MCU activity under resting conditions (Patron et al., 2014). Accordingly, in the PO-induced mouse failing hearts, increased Ca^2+^ transient amplitude induced by urokinase-type plasminogen activator receptor (uPAR) signaling facilitates MCU-mediated Ca^2+^ uptake and increases mitochondrial Ca^2+^ loading (**Figure 2**) (Kanno et al., 2016; Yu et al., 2025). Mitochondrial Ca^2+^ functions as a feed-forward signal that tightly couples cellular energy demand to ATP supply. Ca^2+^ directly activates key enzymes in the TCA cycle, including PDH, isocitrate dehydrogenase (IDH), and oxoglutarate dehydrogenase (Brito-Estrada et al., 2025; Denton et al., 1988; Denton and McCormack, 1990; Glancy and Balaban, 2012; Lee et al., 2023; Territo et al., 2000). This activation increases the substrate flux through the cycle, generating more reducing equivalents (NADH and FADH_2_) to fuel the ETC, thereby driving the proton motive force required for ATP synthesis. However, mitochondrial Ca^2+^ acts as a double-edged sword, exerting detrimental effects when supplied in excess. A pathological increase in mitochondrial Ca^2+^ induces a conformational change in the mitochondrial permeability transition pore that renders it open, leading to collapse of the mitochondrial membrane potential and matrix swelling, and ultimately culminating in cell death (Giorgio et al., 2017; Haworth and Hunter, 1979; Hunter and Haworth, 1979). The upregulation of MCU in the nonischemic human failing hearts and the PO-induced mouse failing hearts increases mitochondrial Ca^2+^ accumulation and is positively correlated with the severity of disease, representing a maladaptive response to heightened energetic demand (Alves-Figueiredo et al., 2024; Zaglia et al., 2017). The coordinated downregulation of MICU3 in failing human hearts serves as a protective mechanism to constrain MCU-mediated Ca^2+^ uptake and mitigate the risk of mitochondrial Ca^2+^ overload (**Figure 2**) (Roman et al., 2024). Furthermore, the protein levels of MICU1 and MICU2, along with the MICU1/MCU ratio, are elevated in the hearts of patients with non-ischemic and ischemic heart failure, enhancing MCU gating by MICU1 and MICU2 and thereby conferring protection against mitochondrial Ca^2+^ overload (Paillard et al., 2022). The ischemic mouse failing hearts employ a distinct mechanism to restrain Ca^2+^ overload by integrating MCUB into the MCU complex (Huo et al., 2020; Lambert et al., 2019). MCUB acts as a dominant-negative paralog of MCU and its incorporation causes displacement of MCU, MICU1, MICU2, and EMRE from the MCU complex, thereby attenuating MCU function and limiting mitochondrial Ca^2+^ uptake (**Figure 2**) (Huo et al., 2020; Lambert et al., 2019; Raffaello et al., 2013). Moreover, in both the human (with ICM) and mouse (with ICM or DCM) failing hearts, the deficiency of dwarf open reading frame (DWORF) microprotein suppresses sarcoplasmic reticulum (SR) Ca^2+^ cycling and reduces peak Ca^2+^ transient amplitude, thereby lowering MCU expression and further constraining mitochondrial Ca^2+^ loading (**Figure 2**) (Brito-Estrada et al., 2025; Makarewich et al., 2020, 2018; Nelson et al., 2016). However, the potential for these adaptive responses to impair TCA cycle activity and oxidative phosphorylation underscores the need for targeted interventions that preserve bioenergetics while limiting mitochondrial damage.

**Figure 2:**
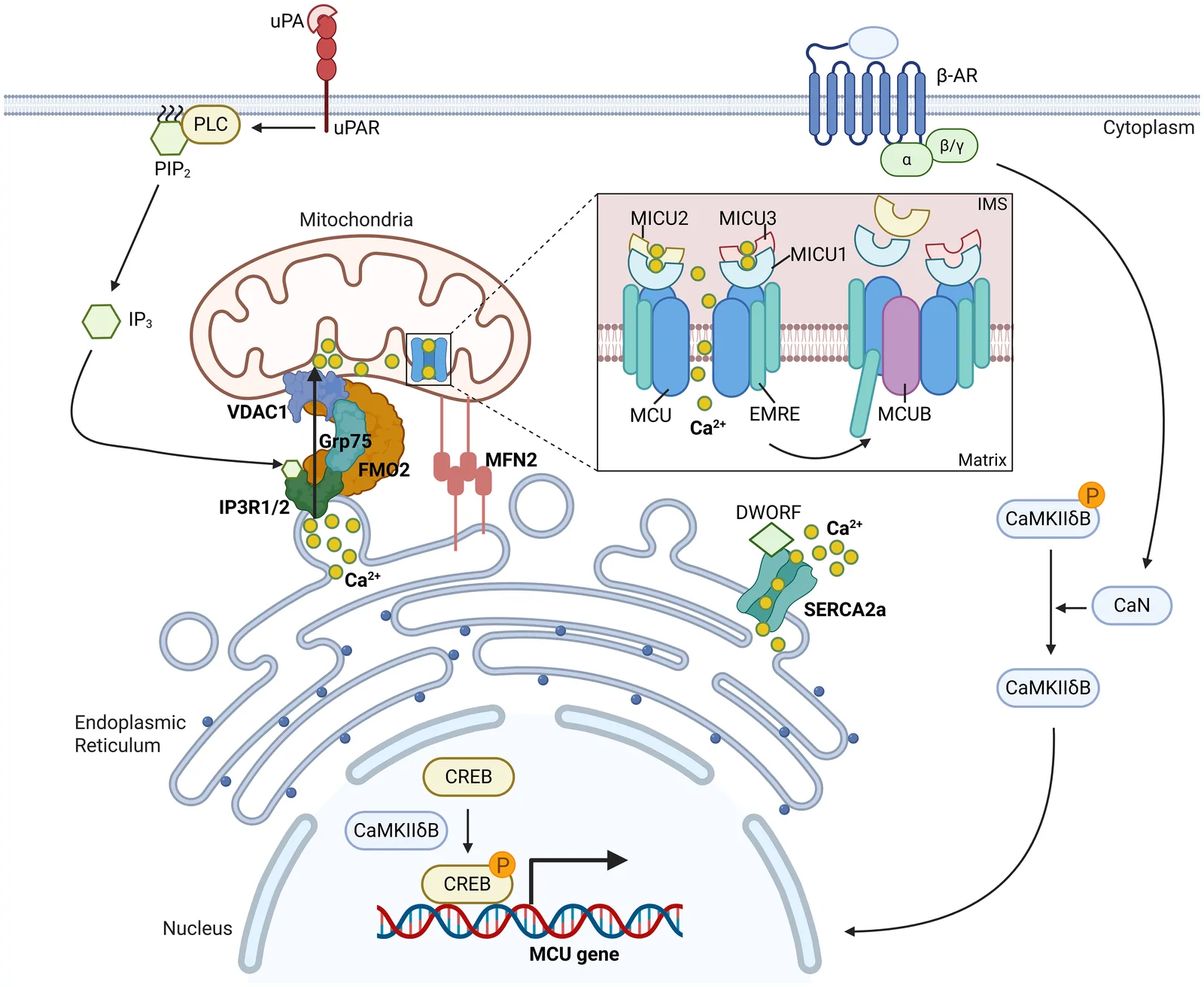
Mitochondrial Ca^2+^ handling in heart failure with reduced ejection fraction. In the pressure overload (PO)-induced mouse failing hearts, increased levels of urokinase-type plasminogen activator receptor (uPAR) promotes activation of phospholipase C (PLC), which cleaves phosphatidylinositol 4,5-bishosphate (PIP_2_) to yield inositol 1,4,5-triphosphate (IP_3_). The binding of IP_3_ to inositol 1,4,5-triphosphate type 1/2 receptor (IP3R1/2) induces Ca^2+^ release from the endoplasmic reticulum (ER), leading to increased mitochondrial Ca^2+^ loading through the mitochondrial Ca^2+^ uniporter (MCU). In the ischemic or PO-induced failing hearts, enhanced β-adrenergic signaling activates β-adrenergic receptor (β-AR), which in turn stimulates calcineurin (CaN) to dephosphorylate CaMKIIδB and facilitate its nuclear translocation. CaMKIIδB then phosphorylates and activates cAMP response element binding protein (CREB) to potentiate MCU expression, which leads to increased mitochondrial Ca^2+^ uptake. Inset: the MCU complex, which consists of MCU, essential MCU regulator (EMRE), mitochondrial Ca^2+^ uptake 1 (MICU1), MICU2, and MICU3. MICU3 potentiates MCU activity, but its downregulation in the human failing hearts leads to reduced mitochondrial Ca^2+^ uptake. In the ischemic mouse failing hearts, MCUB is incorporated into the MCU complex and displaces MCU, MICU1, MICU2, and EMRE from the complex, compromising mitochondrial Ca^2+^ uptake. Dwarf open reading frame (DWORF) microprotein binds to sarco/endoplasmic reticulum Ca^2+^ ATPase isoform 2a (SERCA2a) and enhances its Ca^2+^ transport activity. The downregulation of DWORF in the human (with ICM) and mouse (with ICM or DCM) failing hearts impairs sarcoplasmic reticulum Ca^2+^ cycling and decreases peak Ca^2+^ transient amplitude, ultimately reducing mitochondrial Ca^2+^ uptake. Voltage-dependent anion channel protein 1 (VDAC1), glucose-regulated protein 75 (Grp75), and IP3R1/2 form a tripartite complex that controls ER-mitochondria Ca^2+^ transfer. Flavin-containing monooxygenase 2 (FMO2) acts as a scaffolding protein that facilitates the assembly of VDAC1-Grp75-IP3R2 complex. Mitofusin 2 (MFN2) tethers ER to mitochondria and stabilizes the tripartite complex. In the human failing hearts and the PO-induced mouse failing hearts, the downregulation of FMO2 and MFN2 destabilizes the VDAC1-Grp75-IP3R1/2 complex and impairs mitochondrial Ca^2+^ uptake. IMS, intermembrane space. Created in BioRender. Chen, X. (2026) https://BioRender.com/cr9zusu.

At the whole cell level, mitochondria function as a Ca^2+^ sink that could sequester excessive Ca^2+^, stabilize aberrant Ca^2+^ fluctuations, and maintain cellular Ca^2+^ homeostasis. Chronic β-adrenergic signaling upregulates MCU transcription, enhancing Ca^2+^ uptake into both the SR and mitochondria and thereby preventing cytosolic Ca^2+^ overload-induced calpain activation and cell death (**Figure 2**) (Wang et al., 2022). Conversely, impaired mitochondrial Ca^2+^ uptake elevates cytosolic Ca^2+^ levels and increases the incidence of premature Ca^2+^ transients that predispose to arrhythmia. The uncoupling protein-2 (UCP2)-mediated mitochondrial proton leak in the human (with ICM) and mouse (with ICM or PO-induced) failing hearts decreases mitochondrial membrane potential, reduces MCU-mediated Ca^2+^ uptake, and increases Ca^2+^ spark activity, establishing a mechanistic link between mitochondrial dysfunction and arrhythmogenesis (Eisner et al., 2013; Gaussin et al., 2003; Ji et al., 2015; Murray et al., 2004; Turner et al., 2010). The upregulation of UCP2 in the indicated models is considered maladaptive, despite its constraining effect on mitochondrial Ca^2+^ overload. Accordingly, decreased mitochondrial Ca^2+^ uptake, while protective in inhibiting mPTP opening and preventing cell death, concurrently increases arrhythmogenic potential. Therapeutic strategies designed to prevent mitochondrial Ca^2+^ overload should therefore target both mitochondria and the SR to limit mitochondrial Ca^2+^ loading while enhancing the Ca^2+^ buffering capacity of the cell.

### 5.2 Mitochondrial Ca^2+^ uptake through the VDAC1/Grp75/IP3R1/2 complex

A tripartite complex consisting of voltage-dependent anion channel protein 1 (VDAC1), glucose-regulated protein 75 (Grp75), and inositol 1,4,5-trisphosphate type 1/2 receptor (IP3R1/2) locates at the mitochondria-associated endoplasmic reticulum membranes (MAMs) and controls Ca^2+^ transfer from endoplasmic reticulum to mitochondria (**Figure 2**). Flavin-containing monooxygenase 2 (FMO2) acts as a scaffolding factor that enhances VDAC1-Grp75-IP3R2 interactions and promotes tripartite complex assembly (**Figure 2**) (Xiao et al., 2025). The downregulation of FMO2 in the human (with ICM) and mouse (PO-induced) failing hearts disrupts the integrity of the VDAC1/Grp75/IP3R2 complex and diminishes mitochondrial Ca^2+^ uptake (Ni et al., 2022; Xiao et al., 2025). Mitofusin 2 (MFN2) further stabilizes the tripartite complex by consolidating SR-mitochondria tethering (**Figure 2**) (Chen et al., 2012; Paillard et al., 2013). MFN2 deficiency in the human (with DCM) and mouse (PO-induced) failing hearts triggers SR-mitochondria uncoupling, attenuates SR-mitochondria Ca^2+^ transfer, and ultimately compromises bioenergetics (**Figure 2**) (Mallilankaraman et al., 2012a; Svagusa et al., 2023; Williams et al., 2015; Xin et al., 2019). Mitochondrial tumor suppressor 1A (Mtus1A) joins FMO2 and MFN2 as a key regulator of the VDAC1/Grp75/IP3R1/2 complex, and its deficiency in the ischemic human and mouse failing hearts contributes to impaired mitochondrial Ca^2+^ uptake and respiratory dysfunction (Gong et al., 2025). Genetic deletion of FMO2, MFN2, or Mtus1A exacerbates cardiac dysfunction and injury in response to stress, indicating that these proteins confer protection in heart failure. Accordingly, their downregulation in the indicated heart failure models is regarded as maladaptive.

### 5.3 Na^+^/Ca^2+^ exchanger-mediated mitochondrial Ca^2+^ export

Cytosolic Na^+^ concentration is elevated in human failing hearts with ischemic or dilated cardiomyopathies and animal models of PO-induced heart failure, driving mitochondrial Na^+^ influx through the mitochondrial Na^+^/Ca^2+^ exchanger (mNCE) (Baartscheer et al., 2003; Despa et al., 2002; Liu et al., 2014; Liu and O’Rourke, 2008; Pieske et al., 2002). Accelerated mitochondrial Ca^2+^ efflux is concomitantly induced through the mNCE, lowering mitochondrial Ca^2+^ levels and diminishing the activity of TCA cycle dehydrogenases (Kohlhaas et al., 2010; Maack et al., 2006; Maack and O’Rourke, 2007). However, transgenic NCE overexpression prevents mitochondrial Ca^2+^ overload and attenuates cell death in both PO- and ischemia-induced mouse heart failure models, supporting a protective role for augmented mNCE activity in limiting pathogenic Ca^2+^ accumulation (Garbincius et al., 2022; Luongo et al., 2017).

## 6 Exacerbated oxidative stress in HFrEF

Oxidative stress arises when reactive oxygen species (ROS) production overwhelms the scavenging capacity of endogenous antioxidant defenses. Enhanced activity of ROS-generating enzymes, coupled with compromised antioxidant function, constitutes the primary drivers of oxidative stress, inflicting substantial oxidative injury that contributes to heart failure pathogenesis (Kiyuna et al., 2018; Peoples et al., 2019; van der Pol et al., 2019). In this section, I synthesize recent evidence on the signaling cascades that remodel mitochondrial ROS-producing sites, alongside the transcriptional reprogramming that perturbs the mitochondrial antioxidant apparatus in HFrEF, and elucidate how these perturbations culminate in oxidative damage that disrupts both mitochondrial and cellular homeostasis.

### 6.1 Mitochondrial production of ROS that triggers cellular dysfunction

The mitochondrial ETC is a principal source of cellular ROS, with electron leakage from multiple ETC-associated sites yielding superoxide and hydrogen peroxide, which are key mediators of oxidative damage (Wong et al., 2017). The differential modulation of ROS production sites in heart disease models highlights the complexity of oxidative stress, where specific ETC sites may be restrained for adaptive responses or dysregulated to exacerbate damage. Hyperacetylation-induced ATP synthase inhibition and electron transport defects are upstream drivers of mitochondrial ROS emission in the human failing hearts and a mouse model of PO- or ischemia-induced heart failure (**Figure 3**) (Boudina et al., 2007; Chouchani et al., 2014; Zhang et al., 2018). Elevated mitochondrial membrane potential induced by ATP synthase dysfunction disrupts the normal electron flow through the ETC complexes, causing electron leak to oxygen and superoxide production. Uncoupling protein 3 (UCP3) induces proton leak and lowers mitochondrial membrane potential, which attenuates electron transport defects and limits ROS production. However, the protective effects of UCP3 in ROS limitation are abrogated by Gαq signaling, which downregulates UCP3 expression, reduces proton leak, and drives additional ROS production under ischemic and hypertensive conditions (**Figure 3**) (Azzu et al., 2010; Divakaruni and Brand, 2011; Mailloux et al., 2011; Westenbrink et al., 2015). Mitochondrial complex II, which catalyzes succinate oxidation, emerges as another ETC site that facilitates ROS production. In both the human and mouse hearts, I/R injury leads to substantial succinate accumulation. The rapid oxidation of succinate during reperfusion fuels ROS production through reverse electron transport at mitochondrial complex I (**Figure 3**) (Chouchani et al., 2016, 2014; Yin et al., 2021). Since these ETC-associated alterations potentiate ROS production and elicit oxidative damage, they are regarded as maladaptive. Conversely, the negative regulation of selective ROS sources in response to I/R injury may represent an adaptive response to limit oxidative stress and protect against further mitochondrial damage. Glycerol-3-phosphate dehydrogenase (GPD2) is an integral ETC component and a potent source of ROS (**Figure 3**) (Mráček et al., 2014). MicroRNA(miR)-210-mediated GPD2 downregulation in the mouse hearts following I/R injury limits ROS formation and increases coupling efficiency of mitochondrial energy production (**Figure 3**) (Song et al., 2022). Moreover, I/R-induced downregulation of NADH:ubiquinone oxidoreductase complex assembly factor 2 (NDUFAF2) in rat cardiomyoblasts impedes mitochondrial complex I assembly and reduces complex I-derived ROS production, albeit at the expense of oxidative phosphorylation efficiency (Mohsin et al., 2019; Zhang et al., 2021, 2018).

**Figure 3:**
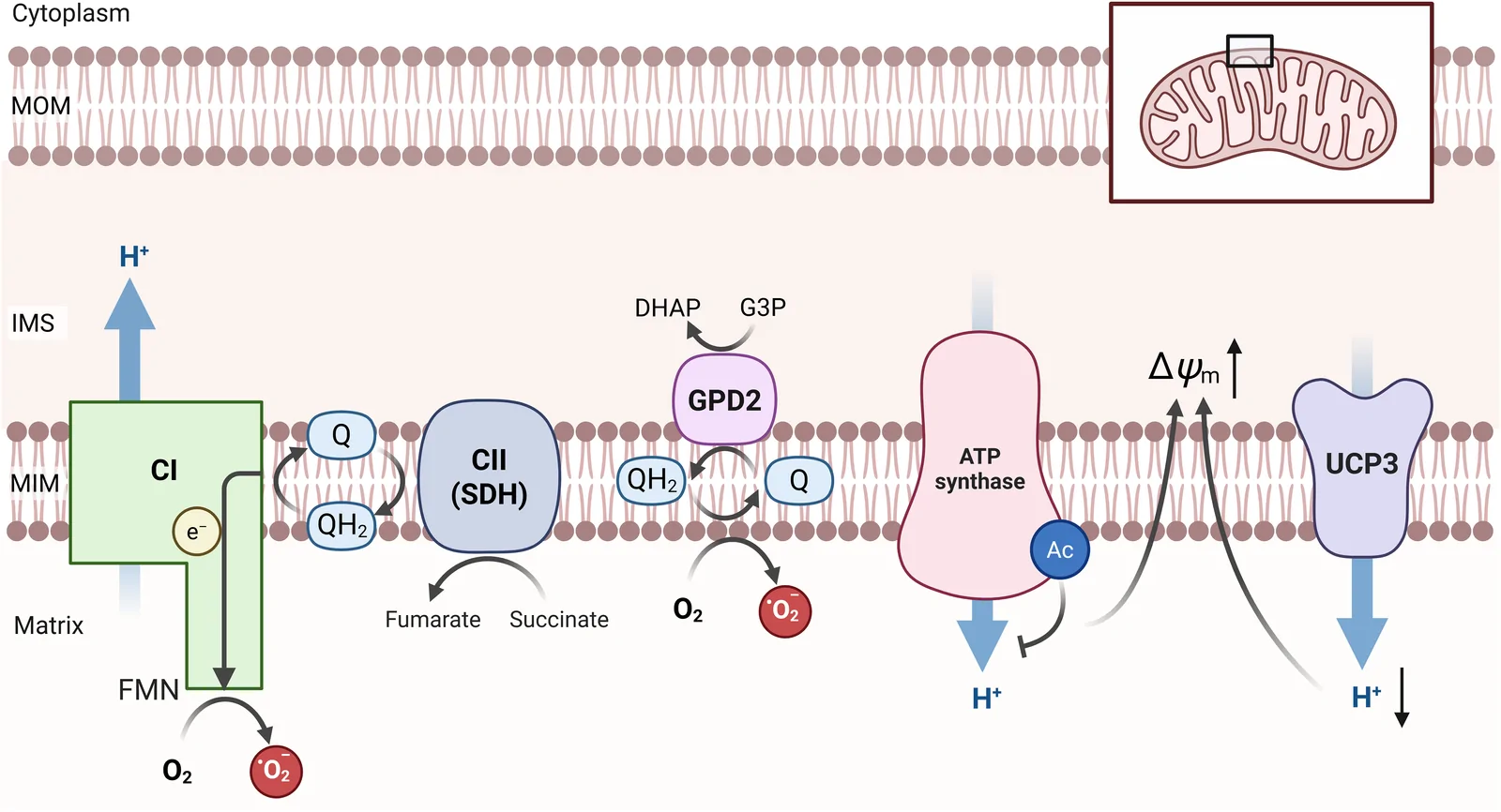
Modulation of electron transport chain (ETC)-associated ROS production sites in heart failure with reduced ejection fraction. Ischemia/reperfusion (I/R)-induced succinate accumulation and rapid oxidation at complex II (CII) reduces ubiquinone (Q) to ubiquinol (QH_2_), which drives reverse electron flow (black arrow) through complex I (CI) onto flavin mononucleotide (FMN). Reduced FMN then delivers electrons to oxygen to induce superoxide formation. Glycerol-3-phosphate dehydrogenase (GPD2) facilitates the conversion of glycerol-3-phosphate (G3P) to dihydroxyacetone phosphate (DHAP) while reducing Q to QH_2_. QH_2_ then delivers electrons to oxygen to induce superoxide formation. MicroRNA-210-mediated GPD2 downregulation during I/R reduces superoxide formation in the mouse hearts. In both the human failing hearts and the mouse model of ischemic or PO-induced heart failure, hyperacetylation (Ac)-induced inhibition of ATP synthase reduces proton influx into the mitochondrial matrix. Moreover, Gαq signaling-induced downregulation of uncoupling protein 3 (UCP3) reduces proton leak. All of these alternations contribute to elevated mitochondrial membrane potential (Δ*ψ*_m_), which impairs electron transport and induces electron leakage and superoxide formation. SDH, succinate dehydrogenase; MOM, mitochondrial outer membrane; IMS, intermembrane space; MIM, mitochondrial inner membrane. Created in BioRender. Chen, X. (2026) https://BioRender.com/5y8hgvo.

NADPH oxidase (NOX) proteins, particularly the mitochondrial isoform NOX4, represent a non-ETC source of ROS that is being increasingly recognized for its role in redox signaling and pathology (Bedard and Krause, 2007; Serrander et al., 2007). NOX4 expression is upregulated in both the human (with ICM or DCM) and mouse (with ICM or PO-induced) failing hearts, contributing to elevated ROS production (Ago et al., 2010; Fraccarollo et al., 2011; Varga et al., 2017; Zeng et al., 2020). In ischemic mouse failing hearts, increased NOX4 expression is partially attributable to mineralocorticoid receptor signaling (Fraccarollo et al., 2011). Monoamine oxidase (MAO), primarily anchored to the outer mitochondrial membrane, constitutes an additional ROS source. In human ischemic failing hearts and a mouse model of PO-induced heart failure, the expression of both MAO isoforms (MAO-A and B) are elevated, leading to enhanced ROS generation and ventricular dysfunction (Kaludercic et al., 2010; Manni et al., 2016). p66Shc promotes hydrogen peroxide production by catalyzing electron transfer from cytochrome *c* to oxygen (Di Lisa et al., 2017). In the pig hearts subjected to I/R injury, p66Shc is phosphorylated and translocated to mitochondria, where it exerts deleterious redox effects under conditions of prolonged ischemia (Yang et al., 2014). p66Shc protein levels are not increased in the hearts of patients with ischemic heart failure (Baysa et al., 2015). Whether human heart failure employs a comparable post-transcriptional mechanism for p66Shc regulation has yet to be established. These alterations in non-ETC source of ROS are maladaptive since they add to the burden of oxidative stress.

In the failing myocardium, excessive ROS production from mitochondria induces adverse protein remodeling and can be associated with a broad spectrum of pathologies. In the human and mouse models of hypertrophic cardiomyopathy (HCM), hypercontractility-induced oxidative modification of mitochondrial creatine kinase (Mt-CK) impairs cytosolic ATP regeneration, potentiates mitochondrial ROS production, and sustains a cycle of redox imbalance (Schlattner et al., 2006; Xu et al., 2025; Yoshizaki et al., 1990). Mitochondrial ROS derived from the ETC hampers the activity of cardiac sodium channel (Na_v_1.5), causes conduction block, and increases arrhythmic risk in human heart failure models (Liu et al., 2010; Makielski and Farley, 2006; Valdivia et al., 2005). Furthermore, NOX4-mediated oxidation of the TCA cycle, ETC, and mitochondrial permeability transition pore (mPTP) components causes energy depletion and triggers cell apoptosis in the mouse failing hearts (Ago et al., 2010). Proteome analysis of a pig model of PO-induced heart failure demonstrates that mitochondrial ROS markedly reduces the abundance of nuclear receptors that regulate metabolic gene expression, including PPAR, RXR, and liver X receptor (LXR), pointing to redox-dependent regulation of substrate metabolism (Dey et al., 2018). Notably, some oxidation products can themselves oxidize and inactivate substrate proteins. This feedforward mechanism establishes a self-amplifying cycle of oxidative damage that may sustain injury even after the initial insult subsides. For instance, patients with DCM or right ventricular failure exhibit accumulation of lipid peroxidation product 4-hydroxynonenal (4HNE), which induces adduction of ETC complex proteins and results in mitochondrial respiratory dysfunction (Hwang et al., 2020; Sharma et al., 2020). These maladaptive oxidative modifications cause severe impairment of protein function, culminating in disturbed cellular homeostasis.

Despite their pathogenic role at excessive levels, modest ROS production exerts protective effects by preventing mitochondrial depolarization, improving respiratory function, and promoting mitophagy in MFN2 deficient mice (Song et al., 2014). Mitochondrial ROS also serves essential signaling functions by activating the extracellular signal-regulated kinase (ERK)/MAPK, cytoskeletal remodeling, Rho GTPase, and integrin signaling pathways (Dey et al., 2018). Additionally, moderate levels of ROS derived from NOX2 or NOX4 engages adaptive mechanisms in the mouse hearts following I/R injury by inducing hypoxia inducible factor-1α (HIF-1α) expression and lowering PPARα levels so as to prevent triglyceride deposition and lipotoxicity (Matsushima et al., 2013). These findings argue against aggressive antioxidant therapy in settings where ROS serve protective signaling functions, advocating instead for strategies that balance redox control with preservation of adaptive ROS signaling.

### 6.2 The antioxidative response to elevated pathological stress

The mitochondrial antioxidant machinery functions through a two-step cascade: superoxide dismutase 2 (SOD2) dismutates superoxide, and peroxiredoxin/glutathione peroxidase detoxify the resulting hydrogen peroxide (Forman et al., 2009; Lu and Holmgren, 2014). Hydrogen peroxide decomposition requires that glutathione and thioredoxin are maintained in their reduced state, and this process needs NADPH (Balaban et al., 2005; Ma et al., 2007; Xiao et al., 2018; Ying, 2008). Therefore, pathways and factors that potentiate NADPH production are believed to have a protective effect to attenuate and counterbalance the deleterious action of ROS. In the failing myocardium, excessive ROS production elicits antioxidative responses, achieved in part through acceleration of these NADPH-producing reactions. In clinical models, DCM- or ischemia-induced heart failure induces an antioxidant adaptation whereby the pentose phosphate pathway (PPP), an NADPH-generating shunt of glycolysis, is fueled by β3-adrenergic receptor (β3AR)-directed, elevated glucose influx (Germack and Dickenson, 2006; Michel et al., 2025; Moniotte et al., 2001). The supply of mitochondrial NADPH is also dependent on nicotinamide nucleotide transhydrogenase, NADP^+^-dependent IDH, and NADP^+^-dependent malic enzyme, which all derive their substrates from the TCA cycle (Ying, 2008). Accordingly, increased mitochondrial Ca^2+^ accumulation, which elevates the turnover rate of the TCA cycle, sustains NADPH synthesis and lowers mitochondrial ROS levels in the ischemic or PO-induced failing hearts (Brandes and Bers, 1997; Maack and O’Rourke, 2007). However, since these NADPH-producing reactions divert substrate metabolism away from generating NADH for ATP synthesis, these compensatory responses compromise cardiac energetics and turn out to be maladaptive.

Conversely, cellular responses aimed at mitigating energy deficit in the failing hearts deplete antioxidant reserve and lead to redox imbalance. Increased anaplerosis through carboxylation of pyruvate to form malate consumes NADPH, disrupts the antioxidant system in the human (ICM) and rat (PO-induced) failing hearts, and is considered maladaptive (Lahey et al., 2018; Pound et al., 2009; Sack, 2009; Sorokina et al., 2007). Furthermore, the failing hearts exhibit impaired expression and activity of key antioxidant proteins, culminating in redox perturbation. NAD^+^ serves as a precursor for the phosphorylated dinucleotide pair NADP^+^/NADPH and therefore plays a pivotal role in ROS detoxification. However, the expression of nicotinamide mononucleotide adenylyl transferase (NMNAT), which catalyzes NAD^+^ biosynthesis, is markedly suppressed in both the human and mouse (with DCM or PO-induced) failing hearts, resulting in reduced NADPH production and impaired ROS scavenging capacity (Diguet et al., 2018). Nuclear factor erythroid 2-related factor 3 (NFE2L3), unlike NFE2L2, participates in redox signaling regulation by suppressing antioxidant gene expression and promoting NOX4 gene transcription (Chowdhury et al., 2009; Pepe et al., 2010; Toki et al., 1997). In the infarcted mouse and human hearts, NFE2L3-mediated downregulation of paired-like homeodomain transcription factor 2 (Pitx2) leads to a subsequent decrease in SOD2, glutathione peroxidase-4 (GPX4), and peroxiredoxin 2 protein abundance (Chen et al., 2025; Tao et al., 2016). Fork-head box protein O (FOXO1/3) function as transcriptional activators of key antioxidant enzymes, serving as a critical defense mechanism against oxidative stress. The SIRT1 deficiency in the post-infarct mouse myocardium and human ischemic failing hearts culminates in FOXO1/3 hyperacetylation and functional impairment, compromising the antioxidant transcriptional program that includes SOD2 and catalase (Alcendor et al., 2007; Brunet et al., 2004; Hsu et al., 2010; Yang et al., 2024; Yu et al., 2021). This SIRT1 deficiency, however, is confined to the ischemic heart failure model, as SIRT1 is upregualted in the PO-induced mouse failing hearts (Van Le et al., 2022). Moreover, in both the human and mouse ischemic failing hearts, hypoxia induces phosphorylation-dependent inactivation of IDH2, curtailing NADPH production and weakening the cell’s antioxidative capacity (Fazal et al., 2017; Ying, 2008). IDH2 expression is also downregulated in the hearts of patients with DCM and the PO-induced mouse failing hearts, contributing to elevated oxidative stress (ElBeck et al., 2024; Van Le et al., 2022). Since all of these antioxidant-related alterations compromise ROS scavenging activity and precipitate oxidative stress, they are regarded as maladaptive. Although thioredoxin 2 is downregulated in DCM-affected human failing hearts, promoting mitochondrial ROS production, the concomitant elevation of SOD2 and GPX1 in these failing hearts points to a coordinated antioxidant response aimed at alleviating oxidative stress and restoring redox homeostasis (Ahuja et al., 2013; Huang et al., 2015; Zhang et al., 2004).

## 7 Regulation of mitochondrial permeability transition pore opening in HFrEF

Mitochondrial permeability transition describes a sudden increase in inner mitochondrial membrane permeability to solutes, mediated by the opening of the mPTP, which permits the passage of molecules up to 1.5 kDa. Although the molecular identity and activation mechanisms of mPTP have been well characterized, the remodeling of its regulatory machinery in the context of HFrEF has yet to be reviewed (Halestrap and Pasdois, 2009; Kwong and Molkentin, 2015). This section will therefore delineate the remodeling of both positive and negative mPTP regulators during HFrEF and identify the signaling pathways and factors that orchestrate these changes.

mPTP is a voltage-gated, high-conductance channel localized in the inner mitochondrial membrane (**Figure 4**) (Bernardi, 1999; Crompton, 1999). Prolonged mPTP opening is detrimental to the heart since it causes collapse of mitochondrial membrane potential, ATP depletion, rupture of mitochondrial outer membrane, release of cytochrome *c*, and eventually cell death (Petronilli et al., 2001). Mitochondrial membrane potential decline and oxidative stress are primary drivers of mPTP opening. Accordingly, maladaptive changes that impair oxidative phosphorylation or disrupt redox balance underlie increased mitochondrial membrane permeability. Excessive mitochondrial Ca^2+^ accumulation serves as an additional trigger for mitochondrial membrane permeability transition, linking Ca^2+^ overload to mitochondrial dysfunction and cell death (**Figure 4**) (Giorgio et al., 2017; Haworth and Hunter, 1979; Hunter and Haworth, 1979). Under hypoxia-reoxygenation (HR), the activation of exchange proteins directly activated by cAMP 1 (Epac1) in the mouse cardiomyocytes stabilizes the VDAC1/GRP75/IP3R1 complex and promotes excessive Ca^2+^ transfer to mitochondria, resulting in mitochondrial Ca^2+^ overload and mPTP opening (**Figure 4**) (de Rooij et al., 1998; Fazal et al., 2017; Kawasaki et al., 1998). The expression of Epac1 is upregulated in both the human (with DCM or ICM) and rat (PO-induced) failing hearts, increasing the proclivity of mPTP opening through similar mechanisms (Fazal et al., 2017; Métrich et al., 2008). In the human (with DCM) and mouse (with ICM or PO-induced) heart failure models, G protein-coupled receptor kinase 2 (GRK2) sensitizes the mPTP to Ca^2+^, magnifying the deleterious effects and aggravating cardiac damage (Brinks et al., 2010; Chen et al., 2013; Dzimiri et al., 2004; Huang et al., 2011; Koch et al., 1995; Rockman et al., 2002). Furthermore, the expression of phospholipase A_2_γ (iPLA_2_γ) is moderately increased in the human failing hearts, leading to elevated production of toxic hydroxyeicosatetraenoic acids (HETEs) that predispose the heart to Ca^2+^-induced mPTP opening (Moon et al., 2018). Since these Ca^2+^-related mitochondrial alterations increase the propensity for mitochondrial membrane permeability transition, they are regarded as maladaptive.

**Figure 4:**
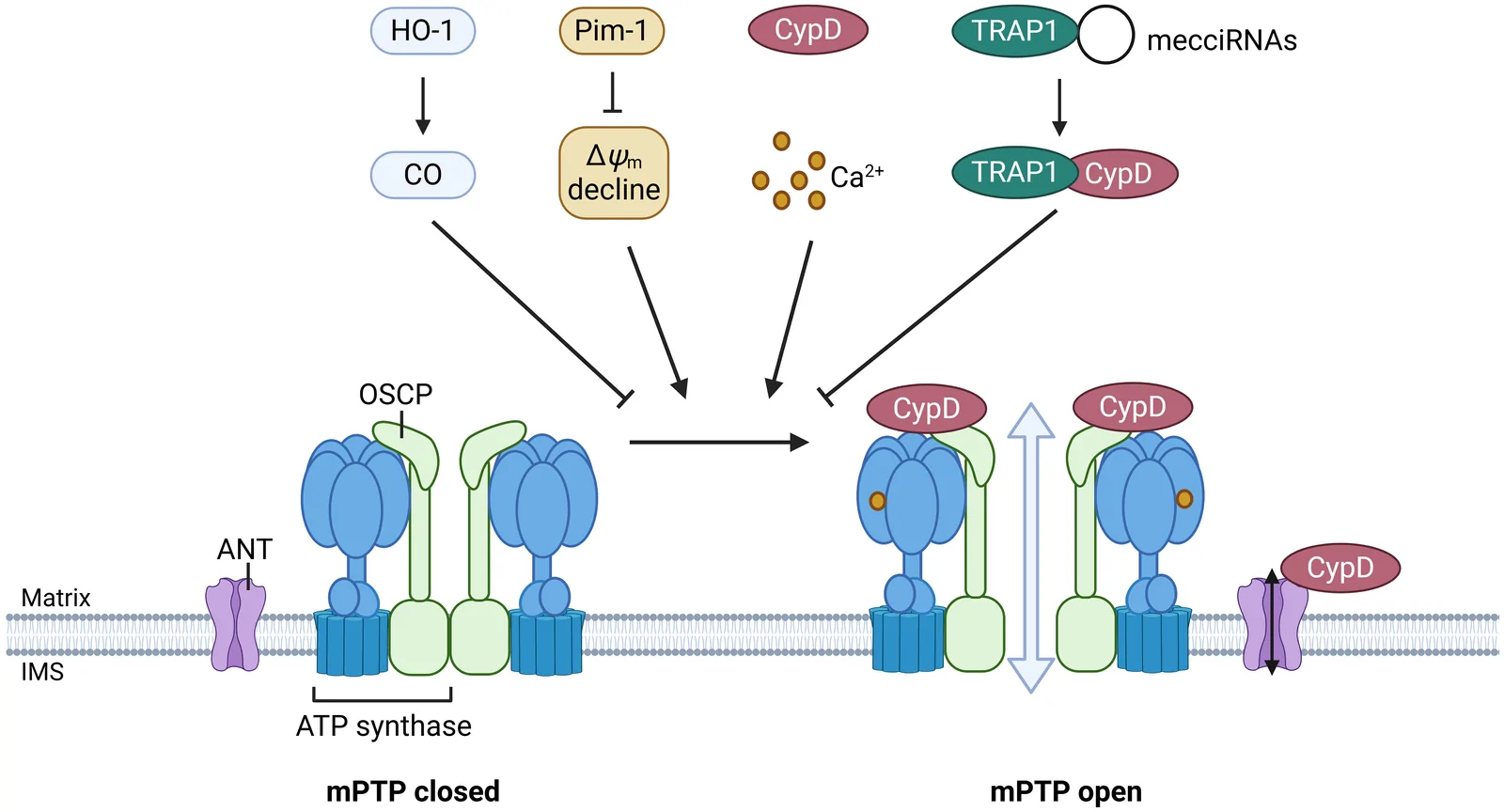
Regulation of mitochondrial permeability transition pore (mPTP) opening in heart failure with reduced ejection fraction. The ATP synthase self-assembles to form a dimer structure, and the mPTP channel formation occurs at the monomer-monomer interface. The adenine nucleotide transporter (ANT) also forms the basic unit of the mPTP. In the human (with DCM or ICM) and mouse (with ICM) failing hearts, elevated expression levels of heme oxygenase-1 (HO-1) drive carbon monoxide (CO) production, which suppresses mPTP opening. In response to ischemia/reperfusion (I/R), mitochondrial translocation of Pim-1 is enhanced, which prevents decline in mitochondrial membrane potential (Δ*ψ*_m_) and inhibits mPTP opening. Mitochondrial Ca^2+^ binds to ATP synthase, causes deformation of the monomers, and induces mPTP opening. In the human (with DCM or ICM), mouse (I/R-induced), and rat (PO-induced) failing hearts, increased ER-mitochondria Ca^2+^ transfer through the VDAC1/Grp75/IP3R1/2 complex causes mitochondrial Ca^2+^ overload and induces mPTP opening. Cyclophilin D (CypD) binds to oligomycin sensitivity-conferring protein (OSCP) and ANT to induce mPTP opening. In the human (with DCM or ICM) and mouse (PO-induced) failing hearts, increased acetylation of CypD enhances its binding affinity to OSCP and promotes mPTP opening. Mitochondria-encoded circular RNAs (mecciRNAs) interact with and maintain high levels of TNF receptor-associated protein 1 (TRAP1), which inhibits CypD from binding to ATP synthase and therefore suppresses mPTP opening. In the DCM-affected human failing hearts and PO-induced mouse failing hearts, mecciRNAs are degraded, leading to enhanced association between CypD and ATP synthase and increased mPTP opening. IMS, intermembrane space. Created in BioRender. Chen, X. (2026) https://BioRender.com/v3kyjf6.

Cyclophilin D (CypD) serves as a positive regulator of mitochondrial membrane permeability transition, acting through its association with ANT and mitochondrial ATP synthase, which are integral to the mPTP (**Figure 4**) (Crompton et al., 1988; Giorgio et al., 2013, 2009; Halestrap and Davidson, 1990). Tumor necrosis factor receptor-associated protein 1 (TRAP1), a negative regulator of mPTP, binds to CypD to block its association with ATP synthase (**Figure 4**) (Cannino et al., 2022; Kang et al., 2007; Xiang et al., 2010). The mitochondrial-encoded circular RNAs (mecciRNAs), which maintain high TRAP1 and low CypD levels in mitochondria, are degraded in the DCM-affected human failing hearts and the PO-induced mouse failing hearts, leading to sustained mPTP opening and mitochondrial disorders (**Figure 4**) (Liu et al., 2025). Moreover, in the human (with DCM or ICM) and mouse (PO-induced) failing hearts, increased acetylation of CypD and oligomycin sensitivity-conferring protein (OSCP) potentiates their binding and increases the permeability of the inner mitochondrial membrane (**Figure 4**) (Lee et al., 2016). Beyond its role in mPTP regulation, CypD also interacts with IP3R1 and Grp75 at MAMs. In rat cardiomyoblasts, HR reinforces the association within the CypD/VDAC1/Grp75/IP3R1 complex, enhancing SR-to-mitochondria Ca^2+^ transfer, and precipitating Ca^2+^ overload and cell death (**Figure 4**) (Paillard et al., 2013). These CypD-related alterations are also maladaptive, as they raise the propensity for mPTP opening and cell death.

Pathological stress also engages adaptive mPTP-inhibitory responses, which may reveal endogenous pathways that can be therapeutically harnessed to limit cardiac injury. In both the human (with ICM or DCM) and mouse (with ICM) failing hearts, heme oxygenase-1 (HO-1)-derived carbon monoxide (CO) attenuates mitochondrial membrane permeability transition and contributes to HO-1’s antiapoptotic actions (**Figure 4**) (Grabellus et al., 2002; Wang et al., 2010). Pim-1 kinase also exhibits mPTP-inhibiting properties by upregulating the expression of anti-apoptotic proteins Bcl-X_L_ and Bcl-2 (Borillo et al., 2010). Bcl-X_L_ and Bcl-2 directly prevent inner membrane depolarization, which is a key step that precedes mPTP opening. In the mouse hearts following I/R injury, Pim-1 kinase’s mitochondrial translocation acts as a stress-responsive protective axis, suppressing mPTP opening and mitochondrial swelling to preserve mitochondrial integrity (**Figure 4**) (Borillo et al., 2010). The elevated Pim-1 expression in the human failing hearts points to a conserved mechanism for restraining mitochondrial membrane permeability transition across human and animal models (Muraski et al., 2007).

## 8 Regulation of mitochondrial homeostasis in HFrEF

Maintenance of mitochondrial homeostasis, achieved through the balanced clearance of damaged mitochondria and the biogenesis of new, functional ones, is fundamental to systemic stability and normal cellular function. This homeostasis is orchestrated by distinct yet interconnected molecular machineries governing mitochondrial fission and fusion, biogenesis, and mitophagy. In HFrEF, these mitochondrial behaviors are reprogrammed in an adaptive attempt to counteract cellular stress; however, certain alterations prove maladaptive and disrupt homeostaic balance. Critically, the integrated coordination among mitochondrial dynamics, biogenesis, and mitophagy attenuates these disturbances and supports the preservation of a dynamic cellular equilibrium. This section reviews recent evidence on the remodeling of mitochondrial processes that impact mitochondrial homeostasis in HFrEF, elucidates how their coordinated interplay fosters the maintenance of a cellular balance, and delineates the implications of these remodeling events for disease progression.

### 8.1 Regulation of genes involved in mitochondrial biogenesis

Mitochondrial biogenesis is a complex process involving the coordinated expression of nuclear and mitochondrial genes, lipid membrane and protein synthesis and assembly, and mitochondrial DNA replication. PGC-1α is a crucial regulator of mitochondrial biogenesis and acts through a regulatory cascade comprising ERRα, nuclear respiratory factor 1 and 2 (NRF1 and NRF2), and transcription factor A, mitochondrial (TFAM) (Duncan et al., 2007; Gleyzer et al., 2005; Kelly and Scarpulla, 2004; Lin et al., 2005; Schreiber et al., 2004; Spiegelman and Heinrich, 2004). PGC-1α lies at the nexus of a regulatory network that governs mitochondrial biogenesis, serving as a convergence point for diverse physiological and pathological signals. The transcription factors and epigenetic machinery that regulate PGC-1α expression have been exhaustively reviewed (She et al., 2026). Extending this framework, the present subsection will interrogate the upstream signaling molecules that impinge upon PGC-1α regulation and elucidate how their activity is remodeled during HFrEF progression.

In the diseased myocardium, PGC-1α and its target genes are subject to opposing regulation: NOX4 represses their expression via a redox-dependent mechanism, whereas Pim kinases and KLF9 promote their expression (**Figure 1**). In ischemia-, DCM- or PO-induced failing hearts, the concurrent upregulation of NOX4 and downregulation of Pim kinases and KLF9 converge to suppress PGC-1α expression, inducing biogenesis defects (**Figure 1**) (Ago et al., 2010; Din et al., 2014; Kim et al., 2020; Zhang et al., 2024). Consistent with these regulatory mechanisms, PGC-1α protein levels are significantly decreased in the hearts of patients with ischemic or nonischemic cardiomyopathy and in the mouse (with DCM or PO-induced) failing hearts, thereby suppressing mitochondrial biogenesis (Burke et al., 2016; Garnier et al., 2009; Wang et al., 2018). Given that PGC-1α positively regulates the expression of PTEN-induced putative kinase-1 (PINK1), Parkin, and FUNDC1 to facilitate both ubiquitin- and receptor-mediated mitophagy, the decline in PGC-1α observed in these heart failure models is anticipated to attenuate mitophagic activity (Liang et al., 2020; Liu et al., 2021). The resultant parallel reduction in both mitochondrial biogenesis and mitophagy may represent a coordinated adaptive mechanism that preserves mitochondrial homeostasis despite the prevailing stress. In addition to transcriptional regulation, PGC-1α activity can also be modulated at the post-translational level via protein modifications. In the human (with DCM) and mouse (with ICM) failing hearts, LARP7 deficiency reduces SIRT1 levels, resulting in PGC-1α hyperacetylation and inactivation, which ultimately impairs mitochondrial biogenesis (Yu et al., 2021). Given that these PGC-1α-related remodeling events culminate in impaired PGC-1α function and diminished biogenesis, they are regarded as maladaptive. Human DCM is characterized by elevated expression of PGC-1α and downstream targets, which overrides the repressive action of DYRK1B and instigates aberrant mitochondrial biogenesis (Ahuja et al., 2013; Lin et al., 2005; Zhuang et al., 2022).

### 8.2 Modulation of mitochondrial fusion and fission

The shapes of mitochondria change continually through the combined actions of fission and fusion, which divide and fuse the two lipid bilayers that surround mitochondria. These structural dynamics are intimately associated with adaptive functional responses to physiological and pathological cues, and genetic disruption of key mitochondrial dynamics proteins has been demonstrated to precipitate heart failure (Knowlton et al., 2014; Panchbhai et al., 2026). This subsection will detail the remodeling of mitochondrial fusion and fission regulatory proteins in HFrEF, with particular emphasis on how these alterations influence disease trajectory.

Mitochondrial fusion proceeds through mitofusin 1 (MFN1)- and MFN2-mediated mitochondria tethering and outer mitochondrial membranes fusion, followed by optic atrophy 1 (OPA1)-mediated inner membrane fusion (Dorn, 2015, 2013). Mitochondrial fission involves severing of the parent mitochondrion into two daughter organelles, which is facilitated by dynamin-related protein 1 (DRP1) (Chang and Blackstone, 2010). Under pathological stress, fusion mitigates damage by mixing the contents of malfunctioning mitochondria as a form of complementation, while fission contributes to quality control by segregating damaged mitochondrial components away from the healthy network for clearance. Mitochondrial fission is a highly regulated process involving distinct signatures (Kleele et al., 2021). Peripheral fission, which occurs near the tips of mitochondria, is preceded by signs of damage and results in the shedding of small mitochondria destined for mitophagy. Fission-mediated segregation of dysfunctional mitochondria into depolarized daughter organelles facilitates autophagosomal engulfment and therefore is a prerequisite for mitophagy (Burman et al., 2017; Twig et al., 2008). In contrast, midzone fission at the center of the organelle is associated with the production of new mitochondria, therefore contributing to mitochondrial biogenesis. Despite these functional contributions, excessive fission, characterized by unregulated activity, fragments healthy mitochondria into smaller, more numerous organelles, compromising membrane potential, respiration, and ATP production (Parra et al., 2018). Accordingly, the dynamic interplay between mitochondrial fusion and fission must be finely tuned to adapt to cellular demands and maintain bioenergetic output. The diseased heart, however, tilts mitochondrial dynamics toward fission by manipulating fission/fusion regulators, culminating in mitochondrial fragmentation and dysfunction. In the failing human (with DCM) and mouse (PO-induced) myocardium, the FUN14 domain containing 1 (FUNDC1) deficiency diminishes IP3R2-mediated SR Ca^2+^ release, resulting in reduced activity of cAMP response element binding protein (CREB) and decreased expression of mitochondrial fission 1 protein (FIS1) (Bi et al., 2022; Li et al., 2020; Wu et al., 2017). However, DRP-1 expression is significantly upregulated in the human and rat (with DCM or ICM) failing hearts, driving extensive fission that facilitates mitophagy and biogenesis but simultaneously jeopardizes mitochondrial integrity and energetics (Chaanine et al., 2019; Shaoqing et al., 2022; Yip et al., 2020). Therefore, despite all the benefits conferred, DRP-1 upregulation in the failing hearts is considered maladaptive as the excessive mitochondrial fragmentation induced increases the propensity for cell death. In a mouse model of PO-induced heart failure, reduced mitochondrial localization of DRP-1 suggests that an alternative mechanism may mediate mitochondrial fission (Shirakabe et al., 2016). Conversely, mitochondrial fusion is compromised in the ischemic or DCM-affected human failing hearts, as OPA1 protein is downregulated through post-translational modifications (Chen et al., 2009, 2012; Wai et al., 2015). Additionally, MFN1 is phosphorylated and functionally suppressed by beta II protein kinase C (βIIPKC) in ischemic rat failing hearts, while MFN2 is downregulated in the DCM-affected human failing hearts and the mouse model of PO-induced heart failure, pointing to a common defect in mitochondrial fusion across HFrEF models (Ferreira et al., 2019, 2012; Kim et al., 2020; Svagusa et al., 2023). Beyond its canonical role in fusion, MFN2 serves as a PINK1-phosphorylated receptor for Parkin, thereby facilitating ubiquitin-mediated mitophagy (Chen and Dorn, 2013). The loss of MFN2 in these heart failure models thus compromises PINK1/Parkin-mediated mitophagic clearance, compounding the deficit in mitochondrial turnover. As fusion downregulation exacerbates the imbalance between fusion and fission, these fusion-related alterations disrupt mitochondrial homeostasis and are maladaptive.

### 8.3 Regulation of mitophagy in response to cardiac stress

Mitophagy is a major quality control pathway that selectively targets damaged or superfluous mitochondria for degradation to preserve mitochondrial homeostasis. During mitophagy, mitochondria are sequestered in double-membrane vesicles called autophagosomes and delivered to lysosomes for degradation (Uoselis et al., 2023). Mitophagy pathways and mechanisms have been systematically reviewed, and their dysregulation in ischemic heart disease has been thoroughly documented (Guo et al., 2026; Ravindran and Gustafsson, 2025; Tang et al., 2024; Uoselis et al., 2023; Wang et al., 2023; Xu et al., 2024). Extending this landscape, the present subsection will focus on more recent advances that illuminate the signaling and transcriptional circuits driving mitophagy reprogramming specifically in the context of HFrEF.

Mitophagy is activated in the mouse hearts during early phase of PO but is suppressed thereafter (Shirakabe et al., 2016). The transient nature of mitophagy activation in PO highlights a failure to sustain mitochondrial quality control, leading to impaired respiration and reduced ATP synthesis (Shirakabe et al., 2016). Attenuation of mitophagy during chronic PO is associated with reduced mitochondrial accumulation of DRP1, a small GTPase that mediates mitochondrial fission and promotes mitophagy through ubiquitin- or receptor-mediated pathways (Ikeda et al., 2015; Lee et al., 2011; Shirakabe et al., 2016; Tanaka et al., 2010). AMPKα2 activates mitophagy by phosphorylating PINK1 and engaging the PINK1/Parkin pathway (**Figure 5**) (Kitada et al., 1998; Valente et al., 2004; Wang et al., 2018). In the human (with DCM or ICM) and mouse (PO-induced) failing hearts, the downregulation of AMPKα2 and its functional inhibition by phosphatase of regenerative liver 2 (PRL2) likely converge to attenuate mitophagic flux (**Figure 5**) (Han et al., 2025; Wang et al., 2018). Notably, Parkin polyubiquitinates mitofusins and targets them for proteasomal degradation, ensuring that mitochondria destined for mitophagy are fragmented and fusion is suppressed (Gegg et al., 2010; Tanaka et al., 2010; Ziviani et al., 2010). Thus, the suppression of the PINK1/Parkin pathway in HFrEF is predicted to derepress fusion, preserving a fused mitochondrial network with functional heterogeneity. In parallel, Parkin coordinates biogenesis by promoting the degradation of PARIS, a transcriptional repressor of PGC-1α (Shin et al., 2011). Consequently, reduced PINK1/Parkin activity in HFrEF elevates PARIS levels and attenuates biogenesis, thereby balancing mitochondrial elimination with renewal. However, reduced biogenesis impedes the replacement of damaged mitochondria with new ones, decreasing the proportion of healthy mitochondria and imposing an additional burden on mitochondria homeostasis. Furthermore, transcription factor EB (TFEB), a central mitophagy regulator that sustains autophagic protein expression, is targeted for degradation by speckle-type POZ protein (SPOP) in the human (with DCM) and mouse (with ICM or PO-induced) failing hearts, resulting in the accumulation of damaged mitochondria (**Figure 5**) (Nezich et al., 2015; Song et al., 2021; Wu et al., 2025; Yang et al., 2023). Importantly, TFEB also promotes PGC-1α expression, positioning it as a nexus linking mitochondrial clearance to biogenesis (Ivankovic et al., 2016). Accordingly, TFEB downregulation in these failing hearts is predicted to suppress PGC-1α expression, coupling attenuated mitophagy with diminished biogenesis to sustain an optimal mitochondrial complement. Dysfunctional mitochondria severely perturb cellular homeostasis by aggravating oxidative stress, disrupting Ca^2+^ handling, and triggering sterile inflammation (Liu et al., 2026; Ma et al., 2022). Therefore, the remodeling events described above lead to mitophagy deficiency, compromise cellular functions, and are considered maladaptive.

**Figure 5:**
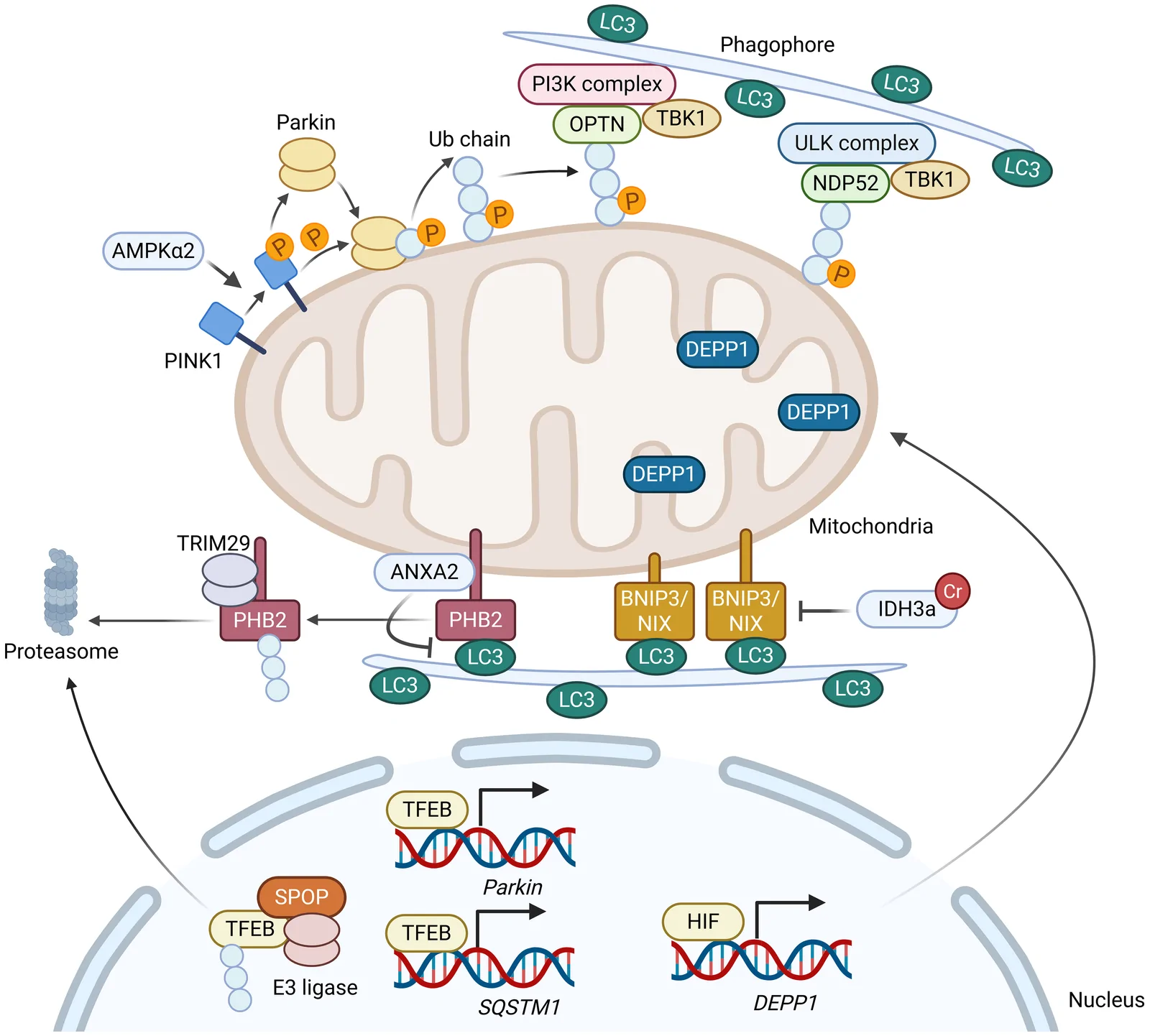
Regulation of mitophagy in heart failure with reduced ejection fraction. Mitophagy is activated by either ubiquitin (Ub)- or receptor-mediated signals. AMP-activated protein kinase subunit α (AMPKα2) interacts with and induces the phosphorylation (P) of PTEN-induced putative kinase-1 (PINK1), which phosphorylates Ub and recruits E3 ubiquitin ligase Parkin to the targeted mitochondria. Parkin further ubiquitinates mitochondrial outer-membrane proteins to amplify the mitophagy signal. Phosphorylated Ub chains recruit autophagy receptor proteins, including optineurin (OPTN) and nuclear dot protein 52 kDa (NDP52), which subsequently recruit autophagosome biogenesis machinery to the targeted mitochondria. The autophagosome biogenesis machinery, including the phosphoinositide 3-kinase (PI3K) complex and the Unc-51 like autophagy-activating kinase (ULK) complex, initiates *de novo* phagophore synthesis at the mitochondria. The phagophore expands and is sealed into a complete autophagosome, which is subsequently delivered to the lysosome for degradation. The human (with DCM or ICM) and mouse (PO-induced) failing hearts exhibit reduced AMPKα2 expression and activity, which attenuates PINK1 activity and impairs PINK1-dependent mitophagy. BCL-2/adenovirus E1B 19-kDa interacting protein 3 (BNIP3) and prohibitin 2 (PHB2) are mitophagy receptors and they recruit microtubule-associated protein 1A/1B light chain 3 (LC3) to initiate autophagosome formation. The lysine crotonylation (Cr) levels of isocitrate dehydrogenase 3 [NAD^+^] alpha (IDH3a) are increased following I/R, which downregulates BNIP3 protein levels and suppresses BNIP3-mediated mitophagy. Annexin A2 (ANXA2) interacts with PHB2, blocks its binding with LC3, and facilitates the proteasomal degradation of PHB2 through E3 ligase TRIM29-mediated polyubiquitination. In ischemic human and mouse failing hearts, ANXA2 upregulation suppresses PHB2-mediated mitophagy. Chronic activation of hypoxia inducible factor (HIF) in human induced pluripotent stem cell-derived cardiomyocytes (hIPS-CMs) elevates the expression of decidual protein induced by progesterone 1 (DEPP1), which localizes inside the mitochondria to promote mitophagy. Transcription factor EB (TFEB) initiates the expression of autophagy-lysosomal and mitophagy-related genes, including *Parkin* and *SQSTM1*. Speckle-type POZ protein (SPOP), the E3 ubiquitin ligase substrate-binding adaptor, interacts with and promotes the ubiquitination and degradation of TFEB. SPOP is upregulated in the human (DCM-affected) and mouse (with ICM or PO-induced) failing hearts, resulting in reduced mitophagic activity. Created in BioRender. Chen, X. (2026) https://BioRender.com/ihc4g5f.

While moderate mitophagy ensures quality control by eliminating dysfunctional mitochondria, sustained mitophagic flux exerts adverse effects by depleting mitochondrial mass, promoting mPTP opening and ROS production, and compromising respiration and bioenergetics. Chronic activation of HIF by hypoxia in the human induced pluripotent stem (iPS) cell-derived cardiomyocytes potentiates the expression of decidual protein induced by progesterone 1 (DEPP1), establishing a persistent mitophagic program that reduces mitochondrial protein abundance and metabolic capacity (**Figure 5**) (Wyant et al., 2024). Genetic deletion of DEPP1 attenuates contractile dysfunction and adverse cardiac remodeling induced by chronic HIF activation, suggesting that hypoxia-induced DEPP1 upregulation is maladaptive (Wyant et al., 2024). Furthermore, increased lysine crotonylation (Kcr) of isocitrate dehydrogenase 3 [NAD^+^] alpha (IDH3a) in the mouse hearts in response to I/R injury restrains BCL-2/adenovirus E1B 19-kDa interacting protein 3 (BNIP3)-mediated mitophagy, preventing mitochondrial depolarization and cell apoptosis (**Figure 5**) (Cai et al., 2022). A Kcr-mimicking mutant of IDH3a preserved post-I/R myocardial function, indicating that suppression of BNIP3-mediated mitophagy is adaptive (Cai et al., 2022). BNIP3 is a established driver of mitochondrial fission, operating through DRP1 recruitment to the outer mitochondrial membrane and direct antagonism of OPA1-mediated fusion (Landes et al., 2010; Lee et al., 2011). The suppression of BNIP3 following I/R injury therefore reorients the dynamics equilibrium toward fusion, preserving an interconnected, less fragmented mitochondrial network. While these evidence indicates excessive mitophagy in the ischemic or I/R-induced failing hearts, other findings corroborate the opposite. In both the human and mouse ischemic failing hearts, annexin A2 (ANXA2) blocks the receptor-mediated mitophagy pathway by inducing the degradation of mitochondrial membrane mitophagy receptor prohibitin 2 (PHB2) (**Figure 5**) (Deng et al., 2026). ANXA2 knockout in MI mice resulted in improved left ventricular function and alleviated cardiac dilation, suggesting that ANXA2-mediated mitophagy suppression is maladaptive (Deng et al., 2026). Moreover, in both the human and rat models of ischemic heart failure, FUNDC1-mediated mitophagy is suppressed by miR-130a, precipitating ischemic injury (Yan et al., 2023). A recent study demonstrates that both autophagy and mitophagy are upregulated in the mouse hearts at 1-7 days after MI (Xu et al., 2022). However, components of the PINK/Parkin mitophagy pathway and the FUNDC1- and BNIP3-mediated mitophagy pathways are downregulated in the human (with DCM or ICM) failing hearts, indicative of suppressed mitophagic activity (Svagusa et al., 2023). One model that reconciles all these findings is that mitophagy in ischemic heart failure proceeds through two phases of regulation, an initial phase of activation followed by a chronic phase of suppression. This helps to explain why contradictory conclusions are obtained from the distinct mitochondrial phenotypes decribed above.

## 9 The role of serum response factor in mitochondrial remodeling in HFrEF

SRF is a transcription factor that governs expression of genes essential for cell growth, cytoskeletal organization, and muscle differentiation in the mammalian heart (Olson and Nordheim, 2010). In neonatal mouse hearts, SRF functions as a positive regulator of cardiomyocyte maturation, driving the expression of genes involved in mitochondrial dynamics, biogenesis, transcription, and oxidative phosphorylation (Guo et al., 2018). In the adult hearts, however, SRF’s role in regulating mitochondrial and metabolic genes switches from stimulatory to inhibitory (Guo et al., 2018). Indeed, cardiac SRF overexpression significantly represses the expression of PGC-1α, PGC-1β, OPA1, and SOD2, resulting in attenuated fatty acid metabolism, reduced mitochondrial biogenesis, suppressed fusion, and impaired antioxidant capacity (Patyal et al., 2025). Moreover, key ETC components are downregulated in SRF transgenic mice, compromising respiratory function (Patyal et al., 2025). In human DCM and ischemic heart failure, SRF undergoes caspase-3-mediated cleavage, generating a dominant-negative form that lacks the transcriptional activation domain (Chang et al., 2003). Concurrently, alternative splicing produces increased levels of a dominant-negative SRF isoform, accompanied by reduced full-length SRF expression in the human failing hearts (Davis et al., 2002). The accumulation of these truncated SRF forms interferes with full-length SRF function, thereby relieving SRF-mediated repression of mitochondrial genes and promoting fatty acid oxidation, mitochondrial biogenesis and fusion, ROS scavenging capacity, and oxidative phosphorylation in the failing heart.

## 10 Mitochondria-mediated inflammatory responses to stress

Mitochondrial DNA that is released into the cytosol and unprocessed by autophagy activates the receptor interacting protein kinase 3 (RIPK3)-NF-κB signaling pathway, eliciting inflammatory responses that exacerbate tissue injury (Broz and Dixit, 2016; Huang et al., 2020; Oka et al., 2012; Sasai et al., 2010; West et al., 2015). The USP10-metaxin 2 (MTX2) axis connects mitochondrial structural integrity to inflammatory signaling, with its disruption in the human (with ICM) and mouse (with ICM or PO-induced) failing hearts propagating a cascade from cristae damage to inflammation (Li et al., 2025; Zhao et al., 2026). Moreover, short transmembrane mitochondrial protein 1 (STMP1) interacts with mitochondrial contact site and cristae organizing complex (MICOS) and sorting and assembly machinery (SAM), which are components of the cristae organizing complexes, to maintain cristae integrity (Ruberto et al., 2026). In the DCM-affected human failing hearts, the downregulation of STMP1 destabilizes mitochondrial cristae and causes mtDNA release into the cytosol, culminating in the stimulation of inflammatory responses (Ruberto et al., 2026). Since inflammatory pathways tend to inflict unintended damage, the downregulation of USP10 and STMP1 is considered maladaptive. As a protective mechanism, Z-DNA binding protein 1 (ZBP1) is upregulated in the human (with ICM) and mouse (with ICM or PO-induced) failing hearts to inhibit RIPK3-NF-κB pathway activity, suppress inflammatory cytokines expression, and prevent infarct expansion (Enzan et al., 2023; Zhang et al., 2025).

## 11 Alternative splicing-induced mitochondrial remodeling

lncRNA dilated cardiomyopathy repressive transcript (DCRT) inhibits third exon skipping of NDUFS2, preventing the generation of a short NDUFS2 isoform that would compromise complex I and peroxiredoxin 5 activity. Accordingly, the downregulation of DCRT in human DCM and the PO-induced mouse failing hearts reduces ATP production and elevates ROS levels, linking splicing dysregulation to bioenergetic failure and oxidative stress (Du et al., 2024). DCRT downregulation in the indicated heart failure models is maladaptive. Furthermore, hypoxia induces a spliced variant of BNIP3 in neonatal rat ventricular myocytes, which interferes with BNIP3’s integration into the mitochondrial membrane and prevents BNIP3 from exerting its pro-apoptotic effects on mitochondria (Gang et al., 2011). Therefore, induction of this alternative splicing event during hypoxia is adaptive.

## 12 Preclinical and clinical therapeutic strategies targeting mitochondrial alterations in HFrEF

While adaptive mitochondrial remodeling alleviates contractile failure and energetic deficits, maladaptive processes contribute significantly to the pathogenesis and progression of heart failure, making mitochondria a pivotal therapeutic target. A wide range of preclinical strategies, from genetic manipulations to pharmacologic interventions, have been explored to restore mitochondrial integrity and cardiac function in animal models of HFrEF. Clinical therapeutic strategies, developed based on preclinical evidence and an understanding of HFrEF pathophysiology, have substantially optimized heart failure management and improved patient survival. This section integrates therapeutic strategies and evidence from both preclinical and clinical studies to provide a translational perspective on the role of mitochondrial remodeling in HFrEF (**Table 2**).

**Table 2:** Mitochondria-targeted therapeutic strategies for HFrEF.

Mitochondrial alternations/rationale	Preclinical strategy	Preclinical evidence	Clinical strategy	Clinical evidence	Current status/limitations
AMPK-PPARα suppression leads to depressed FAO	Genetic deletion of AMPK inhibitor PRL2	Restored FAO, improved cardiac function	Treatment with the PRL2 inhibitor pentamidine		Pentamidine has not been approved for clinical use due to QT prolongation risk
NAD^+^/NADH redox imbalance impairs FAO and oxidative phosphorylation	Enhancing NAD^+^ synthesis via NAD^+^ precursor supplementation (NR or NMN)	Improved FAO and oxidative phosphorylation, preserved contractile function	NR or NMN supplementation		Short-term supplementation increases circulatory NAD^+^ levels, efficacy in improving heart function has not been tested
Increase glycolytic ATP production via inhibiting fatty acid metabolism	Inhibition of malonyl-CoA decarboxylase to restrain mitochondrial fatty acid uptake	Reduced FAO, increased glycolytic flux, improved cardiac function	Reducing free fatty acid availability via adrenergic antagonist carvedilol	COPERNICUS trial: reduced mortality, reduced hospitalization for heart failure	FDA-approved medical therapy for HFrEF, carvedilol has been used in clinical practice for decades
KLF9 and LARP7, positive regulators of PGC-1α, are downregulated	Cardiac-specific overexpression of KLF9 or LARP7	Enhanced FAO and oxidative phosphorylation, improved contractile function, alleviated MI-induced injury	AAV9-based gene therapy for transgenic overexpression of KLF9 or LARP7		AAV9-based gene therapies are available in clinical trials for heart failure, specific trials involving KLF9 or LARP7 have not been initiated
DYRK1B and S100a9, negative regulators of PGC-1α, are upregulated	Treatment with the DYRK1B inhibitor AZ191, genetic deletion of S100a9	Enhanced oxidative phosphorylation, improved cardiac performance	Treatment with DYRK1B inhibitors		All DYRK1B inhibitors are still in preclinical development
METTL4, which interferes with mitochondrial gene transcription, is upregulated	Genetic deletion of METTL4	Preserved ETC gene expression, improved cardiac performance	Treatment with METTL4 inhibitors		Selective METTL4 inhibitors have not been developed for patient use
CaMKIIδ is a repressor of ETC gene expression	Genetic deletion of CaMKIIδ	Reduced respiration defects, mitigated contractile dysfunction	Treatment with the CaMKIIδ inhibitor CRD-4730		CRD-4730 is being tested in a Phase 2 clinical trial for HFrEF
DECR1 and USP10, positive regulators of ETC gene expression, are downregulated	Cardiac-specific DECR1 or USP10 overexpression	Improved mitochondrial energetics and contractile function, reduced cardiac inflammation and infarct size	AAV9-based gene therapy for transgenic overexpression of DECR1 or USP10		AAV9-based gene therapies are available in clinical trials for heart failure, specific trials involving DECR1 or USP10 have not been initiated
H_2_S serves as electron donor for ETC and the availability is diminished	Treatment with H_2_S donor JK-1	Enhanced ETC activity, attenuated contractile dysfunction and cardiac dilation	Treatment with H_2_S donor SG-1002		Phase 1 trial was completed, has not progressed to Phase 2, successfully increased circulating H_2_S levels, safe and well-tolerated at doses up to 800 mg twice daily
uPAR augments mitochondrial Ca^2+^ uptake and is upregulated	Cardiac-specific overexpression of GPLD1, a uPAR inhibitor	Reduced MCU-mediated Ca^2+^ uptake, enhanced cardiac output	Treatment with the uPAR inhibitor WAL0921		Available for clinical trials for kidney diseases, not for heart failure
MCUB is incorporated into the MCU complex and impairs complex integrity	Cardiac-specific overexpression of MCUB	Mitochondrial Ca^2+^ overload prevented, reduced infarct size			
MCU-mediated Ca^2+^ uptake is positively correlated with mitochondrial membrane potential	Mild dissipation of membrane potential via DNP	Restrained mitochondrial Ca^2+^ uptake	Treatment with HU6, which is metabolized to DNP in the liver		HU6 has been evaluated in clinical trials as a potential therapy for HFpEF, but has not been tested in trials for HFrEF
Succinate accumulation and rapid oxidation drives ROS production	Treatment with dimethyl malonate ester, a prodrug of SDH inhibitor malonate	suppressed succinate oxidation, reduced ROS production	Treatment with malonate, a SDH inhibitor with limited toxicity		Malonate remains an experimental compound in the preclinical research phase
ISR, which attenuates oxidative stress, is activated	Salubrinal administration to activate ISR	Reduced ROS production, elevated cardioproection against damage	Salubrinal treatment		No clinical trial has been initiated for Salubrinal
NOX4, a ROS source, is upregulated	Mineralocorticoid receptor ablation	Reduced NOX4 and ROS levels, attenuated contractile dysfunction	Treatment with setanaxib, a NOX4-targeting drug		Setanaxib has not reached clinical development for cardiac indications
β3AR is upregulated to induce NADPH production	Cardiac-specific β3AR overexperssion	Increased NADPH levels, elevated antioxidant capacity	Treatment with mirabegron, a β3AR agonist	Improved cardiac index in HFrEF patients	Mirabegron has completed Phase 2 trial but has not advanced to Phase 3 for heart failure
ME1, which catalyzes anaplerosis that consumes NADPH, is upregulated	Cardiac-specific miR-ME1 overexpression	Attenuated anaplerosis, glutathione level restored, improved cardiac function	AAV9-based gene therapy for transgenic overexpression of miR-ME1		AAV9-based gene therapies are available in clinical trials for heart failure, specific trials involving miR-ME1 have not been initiated
Epac1, which promotes CaMKII-mediated IDH2 inactivation, is upregulated	Treatment with the CaMKII inhibitor KN93	Restored NADPH levels, enhanced antioxidant capacity	Treatment with the CaMKII inhibitor CRD-4730		CRD-4730 is being tested in a Phase 2 clinical trial for HFrEF
NFE2L3, which suppresses antioxidant gene expression, is upregulated	Genetic deletion of NFE2L3	Enhanced ROS scavenging capacity, attenuated contractile dysfunction and cardiac dilation			
LARP7, which enhances antioxidant gene expression, is downregulated by ATM	Treatment with the ATM inhibitor KU60019	Restored antioxidant gene expression, improved systolic function	Treatment with ATM inhibitors		Several ATM inhibitors have entered human trials for cancer therapies, not for heart failure
Coenzyme Q_10_ is downregulated	Coenzyme Q_10_ supplementation	Attenuated cardiac dysfunction and myocardial infarct size	Coenzyme Q_10_ supplementation	Q-SYMBIO trial: reduced cardiovascular mortality, reduced incidence of hospital stays for HF	Coenzyme Q_10_ has progressed to large-scale Phase 3 outcome trials, limited mitochondrial permeability, large doses (2400-3000 mg/day) are required
Coenzyme Q_10_ is downregulated	MitoQ (a modified version of coenzyme Q_10_) supplementation	Increased expression of metabolism-regulated genes, ameliorated left ventricular dysfunction	MitoQ supplementation	Treatment with a dose of 40 mg/day for 12 months failed to induce improvement in myocardial energetics and contractile function in DCM patients	MitoQ is being tested in a Phase 2 trial for DCM
Epac1 is upregulated to induce mitochondrial Ca^2+^ overload and mPTP opening	Treatment with the Epac1 inhibitor CE3F4	Blocked mPTP opening, mitigated cell apoptosis, reduced infarct size	Treatment with Epac1 inhibitors		An Epac1-selective inhibitor for clinical trials is currently unavailable
GRK2, which increases mPTP sensitivity to Ca^2+^, is upregulated	Treatment with the MEK inhibitor PD98059 to prevent GRK2 activation and mitochondrial translocation	Attenuated stress-induced cell death	Treatment with the GRK2 inhibitor paroxetine	CARE-AMI trial: reduced infarct size after 12 weeks of treatment (20 mg/day), but no functional improvement after 1 year of treatment	Paroxetine has been evaluated in Phase 2 clinical trials for heart failure, high dose is associated with stronger off-target effects
mecciRNAs, which inhibit mPTP opening, are downregulated	mecciND2 administration	Suppressed mPTP opening, delayed transition to decompensation			
Enhanced interactions within the CypD/VDAC1/ Grp75/IP3R1 complex induces mitochondrial Ca^2+^ overload and mPTP opening	Treatment with the CypD inhibitor NIM811	Reduced mitochondrial Ca^2+^ levels, attenuated HR-induced cell death	Treatment with the CypD inhibitor NIM811		NIM811 has not entered clinical trials for heart failure therapy
HO-1 is upregulated to inhibit mPTP opening in a CO-dependent manner	Cardiac-specific HO-1 overexpression, treatment with the CO donor CORM-3	Suppressed mPTP opening, attenuated left ventricular dilatation and systolic dysfunction	Treatment with CO donors (CORMs)		CORMs have not entered any human trials for heart failure
Pim-1 is a mPTP inhibitor and its mitochondrial translocation is enhanced	Cardiac-specific Pim-1 overexpression	Suppressed mPTP opening, reduced apoptosis	AAV9-based gene therapy for transgenic overexpression of Pim-1		AAV9-based gene therapies are available in clinical trials for heart failure, specific trials involving Pim-1 have not been initiated
Cyclosporin inhibits mPTP opening through interactions with CypD			Cyclosporin treatment	Pilot Trial: Reduced infarct size, attenuated LV dilation, improved LV ejection fraction with a daily dose of 2.5 mg per kg of body weight for 6 months; CIRCUS Trial: no reduction in mortality, hospitalization, or LV remodeling	Cyclosporin has entered Phase 3 trial for heart failure, exhibiting off-target effects such as immunosuppression and renal pathology, not appropriate for long-term heart failure management
Mitophagy is downregulated and can be potentiated using TB1	Treatment with TB1	Restored mitophagic flux, preserved LV ejection fraction, attenuated cell apoptosis			Displaying unintended side effects, not a appropriate drug for clinical use
The expression and activity of AMPKα2, which activates the PINK/Parkin-dependent mitophagy, are downregulated	Cardiac-specific AMPKα2 overexpression	Enhanced PINK1 and Parkin activity, attenuated adverse cardiac remodeling	Treatment with dapagliflozin, an activator of AMPKα2-dependent mitophagy	DETERMINE trial: improved symptoms with a dose of 10 mg/day for 16 weeks; DAPA-HF trial: reduced risk of worsening heart failure and death from cardiovascular causes with a dose of 10 mg/day for 18 months	FDA-approved medical therapy for HFrEF
SPOP, which promotes TFEB degradation, is upregulated	Treatment with the SPOP inhibitor I-6lc	Restored TFEB levels and expression of autophagy-related genes, improved cardiac function	Treatment with SPOP inhibitors		No SPOP inhibitor has entered clinical trials for heart failure
ANXA2, which mediates the degradation of the mitophagy receptor PHB2, is upregulated	Genetic deletion of ANXA2	Restored PHB2 levels, enhanced mitophagic activity, mitigated LV dilatation and contractile dysfunction	Treatment with ANXA2 inhibitors		An ANXA2 inhibitor is currently not available for clinical trials for heart failure

### 12.1 Therapeutic strategies targeting energetic failure in HFrEF

Metabolic remodeling is a hallmark of human heart failure, with substantial evidence from both patients and animal models implicating mitochondrial alterations in the pathogenesis of myocardial energetic impairment (Lopaschuk et al., 2021). A reduced energetic reserve, reflected by a low phosphocreatine-to-ATP (PCr/ATP) ratio, is frequently observed in heart failure patients and strongly predictive of mortality (de Wit-Verheggen et al., 2023; Neubauer, 2007). Accordingly, therapeutic strategies designed to enhance the energy-demanding contractile performance of the failing heart should target mitochondrial energy production, with a specific focus on the metabolic reprogramming encompassing altered fuel preference and ETC function.

Deranged substrate utilization is a central feature of mitochondrial dysfunction in HFrEF, where impaired FAO contributes to bioenergetic failure. Two distinct but related mechanisms have been implicated. First, suppression of the AMPK-PPARα axis has been causally linked to depressed FAO in the human (with ICM or DCM) and mouse (PO-induced) failing hearts. Genetic deletion of the AMPK inhibitor PRL2 restored fatty acid utilization and improved cardiac function in the mouse model of PO-induced heart failure (Han et al., 2025). Second, chronic NAD^+^/NADH redox imbalance impairs NAD^+^-dependent SDR and sirtuin activity, imposing a substantial burden on FAO, oxidative phosphorylation, and acetylation-associated protein homeostasis in the PO-induced mouse failing hearts and human DCM/ischemic heart failure. Augmenting NAD^+^ synthesis via nicotinamide riboside chloride (NR) or nicotinamide mononucleotide (NMN) treatment re-established SDR and sirtuin activity, improved FAO and mitochondrial respiration, normalized protein acetylation, and preserved contractile function in the mouse failing hearts (Lee et al., 2016; Walker et al., 2023). Despite this promise, clinical translation is impeded by two obstacles: pentamidine, identified as a PRL2 inhibitor, has not secured approval for human use (Pathak et al., 2002), and NR/NMN, while well tolerated and effective at elevating NAD^+^ levels in short-term trials, have not achieved approved pharmaceutical drug status owing to the absence of large, long-term clinical trials (Christen et al., 2026; Gallagher and Emmanuel, 2026).

A reverse therapeutic approach, aimed at inhibiting fatty acid catabolism while augmenting mitochondrial glucose oxidation, has been pursued in both preclinical and clinical settings to address the bioenergetic deficits of the failing heart. Adrenergic antagonists are employed in HFrEF to diminish circulating free fatty acid availability and enhance glycolytic ATP production (Jaswal et al., 2011). Carvedilol, a β-adrenergic receptor antagonist, has been clinically demonstrated to reduce mortality and hospitalization for heart failure (Fowler, 2004; Packer et al., 2002). Furthermore, malonyl-CoA restrains endogenous fatty acid concentrations by inhibiting CPT1, the rate-limiting enzyme for mitochondrial fatty acid uptake (Jaswal et al., 2011). Inhibition of malonyl-CoA decarboxylase, which catalyzes malonyl-CoA degradation, has been reported to reduce FAO, increase glycolytic flux, and improve post-ischemic cardiac function in preclinical models (Dyck et al., 2004). However, studies of malonyl-CoA decarboxylase inhibitors in heart failure patients remain lacking and are warranted.

As a master regulator of fatty acid metabolism, oxidative phosphorylation, and mitochondrial biogenesis, PGC-1α integrates multiple signaling pathways and represents a promising therapeutic target (**Figure 1**). The expression and activity of PGC-1α regulators have been manipulated in preclinical models to improve mitochondrial energetic and biogenic status. KLF9 is downregulated in PO-induced mouse failing hearts and in human DCM- or ischemia-induced heart failure, leading to reduced PGC-1α expression. Transgenic KLF9 overexpression in mouse myocardium elevated PGC-1α expression, enhancing FAO, oxidative phosphorylation, and mitochondrial biogenesis under basal conditions (Zhang et al., 2024). LARP7 is likewise downregulated in the infarcted mouse hearts and the DCM-affected human failing hearts, contributing to reduced PGC-1α activity. In preclinical models, cardiac LARP7 overexpression potentiated PGC-1α activity, attenuated contractile dysfunction, and alleviated MI-induced cardiac injury (Yu et al., 2021). DYRK1B expression is markedly elevated in the PO-induced mouse failing hearts and in the human (with DCM or ICM) failing hearts, suppressing PGC-1α expression. DYRK1B-mediated PGC-1α suppression can be relieved by the DYRK1B inhibitor AZ191, which enhances oxidative phosphorylation and represses pathological remodeling in the mouse heart failure model (Zhuang et al., 2022). S100a9 is a potential therapeutic target in the mouse I/R model, where its deletion derepresses PGC-1α expression and improves cardiac performance (Li et al., 2019). AAV9-based gene therapies are currently under investigation and have been approved for a range of heart diseases (Desai et al., 2025; Greenberg et al., 2025). Therefore, elevating PGC-1α expression and activity via AAV9-mediated KLF9 or LARP7 overexpression holds promise for clinical research aimed at resolving energetic deficits and improving cardiac function in HFrEF. However, DYRK1B-targeted therapy remains challenging, as all DYRK1B inhibitors are still in preclinical development.

ETC gene expression can also be modulated in a PGC-1α-independent manner to enhance oxidative phosphorylation efficiency. METTL4, which increases 6mA abundance within mtDNA and interferes with mitochondrial gene transcription, is upregulated in both the mouse and human heart failure models. Genetic deletion of METTL4 preserved the expression of ETC subunits and improved cardiac performance in the mouse failing hearts (Zhang et al., 2024). Although a METTL4 antagonist has been identified and tested in preclinical models, highly selective inhibitors have not been developed for patient studies, rendering METTL4-targeted therapies challenging (Zheng et al., 2025). Genetic deletion of CaMKIIδ, a repressor of ETC genes, rescued respiration defects and alleviated contractile dysfunction in Gαq transgenic mice (Westenbrink et al., 2015). The CaMKIIδ inhibitor CRD-4730 is currently being evaluated in a Phase 2 clinical trial for HFrEF patients, positioning CaMKIIδ as a promising target for treating ETC-associated energetic failure. DECR1 and USP10, both positive regulators of ETC gene expression, are downregulated in infarcted mouse and human hearts, resulting in oxidative phosphorylation defects. In preclinical studies, DECR1 or USP10 overexpression improved mitochondrial bioenergetics, mitigated contractile dysfunction, and reduced cardiac inflammation and infarct size by increasing ETC gene expression (Duan et al., 2024; Zhao et al., 2026). Despite this promise, clinical translation of this transgenic overexpression strategy depends on the stability and safety of AAV9-based gene therapy. In the ischemic human failing hearts and the PO-induced mouse failing hearts, reduced ETC activity is also attributable to diminished availability of H_2_S, which normally supplies electrons to the ETC to promote oxidative phosphorylation. In the mouse heart failure model, H_2_S therapy with the donor JK-1 enhanced ETC activity and attenuated PO-induced contractile dysfunction and cardiac dilatation (Li et al., 2022). Anethole dithiolethione, a slow-release H_2_S donor, exhibits cardioprotection in preclinical studies, yet its clinical translation for human heart failure remains lacking (Polhemus et al., 2013). SG-1002, an H_2_S donor evaluated in clinical studies, has been investigated for increasing circulating H_2_S to improve heart failure outcomes (Marwah et al., 2026). However, these studies have not progressed to Phase 2 trials, and the translational potential remains elusive.

### 12.2 Therapeutic strategies targeting mitochondrial Ca^2+^ handling in HFrEF

In the human failing hearts, elevated MCU expression drives mitochondrial Ca^2+^ overload, which triggers mitochondrial membrane permeability transition and ultimately cell death (Alves-Figueiredo et al., 2024). Therapeutic strategies aimed at mitigating cardiac injury and dysfunction in HFrEF have focused on targeting mitochondrial Ca^2+^ channels and their regulatory machinery to limit Ca^2+^ loading. uPAR expression is elevated in the PO-induced mouse failing hearts, leading to augmented Ca^2+^ release from the SR and upregulated mitochondrial Ca^2+^ uptake through the MCU. Overexpression of glycosylphosphatidylinositol-specific phospholipase D1 (GPLD1), which mediates uPAR inactivation, reduced MCU-mediated Ca^2+^ uptake and ultimately enhanced cardiac output in these mouse failing hearts (Yu et al., 2025). The uPAR inhibitor WAL0921 is currently available for clinical trials in kidney diseases. However, whether it holds translational potential for human heart failure remains to be established. MCUB functions as a dominant-negative subunit of the MCU complex and is incorporated into the MCU complex in the ischemic mouse failing hearts. In preclinical models, cardiac-restricted MCUB overexpression prevented mitochondrial Ca^2+^ overload and reduced infarct size by approximately 50% (Lambert et al., 2019). However, the functional mechanisms of MCUB in human heart failure models have not been reported, and no MCUB-targeting drugs have entered clinical trials for heart failure, constraining its translation potential. Since MCU-mediated Ca^2+^ uptake is positively correlated with mitochondrial membrane potential, mild dissipation of membrane potential using 2,4-dinitrophenol (DNP) represents a protective strategy to limit Ca^2+^ uptake in preclinical models (Turner et al., 2010). HU6, which is metabolized to DNP in the liver, has been evaluated in humans as a potential therapy for obesity-associated HFpEF (Kitzman et al., 2024). As the correlation between mitochondrial Ca^2+^ uptake and membrane potential is likely conserved across animal and human models, translating DNP treatment into Ca^2+^-targeted therapy in human HFrEF is promising. However, these Ca^2+^-targeted strategies come at a cost: reducing mitochondrial Ca^2+^ loading suppresses the activity of Ca^2+^-sensitive dehydrogenases of TCA cycle and compromises mitochondrial bioenergetics. Treatment parameters should therefore be carefully optimized to attenuate mitochondrial Ca^2+^ overload while preserving moderate Ca^2+^ accumulation sufficient to maintain bioenergetics.

### 12.3 Therapeutic strategies targeting oxidative stress in HFrEF

Increased mitochondrial ROS production has been reported in both the animal and human heart failure models (Belch et al., 1991; Ide et al., 2001; Mallat et al., 1998). Excessive mitochondrial ROS inflicts oxidative damage to a myriad of mitochondrial proteins, posing a substantial threat to multiple mitochondrial functions. Therapeutic interventions have therefore been directed toward either crippling ROS sources or consolidating the antioxidant defense system.

Massive accumulation and oxidation of succinate in ischemic tissue drives mitochondrial ROS production in both preclinical and clinical models (Chouchani et al., 2014; Martin et al., 2019; Prag et al., 2021). In the mouse hearts, pretreatment with dimethyl malonate ester, a prodrug of the succinate dehydrogenase (SDH) inhibitor malonate, prevented succinate oxidation and markedly reduced oxidative stress during I/R (Chouchani et al., 2014; Kula-Alwar et al., 2019). Limited toxicity and a well-established metabolism make malonate an ideal candidate for pharmaceutical therapy. However, high dose requirements, the need for correct timing of administration, and off-target malonate delivery remain obstacles to clinical translation (Kula-Alwar et al., 2019). I/R transiently activates the integrated stress response (ISR) to attenuate oxidative stress in the mouse heart failure models. Pharmacological stimulation of the ISR with Salubrinal maintained high phosphorylation of the α subunit of eukaryotic initiation factor 2 (eIF2α), which enhanced NDUFAF2 turnover, reduced ROS production, and conferred cardioprotection against reperfusion damage in the mouse hearts (Zhang et al., 2021). NOX4, another potent ROS source, is upregulated in the human (with ICM or DCM) and mouse (with ICM or PO-induced) failing hearts due to elevated mineralocorticoid receptor signaling. NOX4-derived ROS can be reduced by mineralocorticoid receptor ablation, which prevents NOX4 upregulation and attenuates MI-induced contractile dysfunction (Fraccarollo et al., 2011). Setanaxib (GKT137831) represents the most advanced NOX4-targeting drug, and its effect in attenuating oxidative stress has been validated in preclinical heart failure models (Labbé et al., 2025). Although under evaluation in clinical trials for other conditions, setanaxib has not reached clinical development for cardiac indications (Yu et al., 2024a).

Therapeutic strategies targeting the antioxidant defense system have demonstrated efficacy in restoring redox balance and myocardial function in preclinical and clinical studies. β3AR expression is elevated in human heart failure models, leading to enhanced NADPH production that alleviates oxidative damage (Moniotte et al., 2001). In preclinical studies, cardiac β3AR overexpression elicited a substantial increase in NADPH levels, elevating the antioxidant capacity of the PO-induced mouse failing hearts (Michel et al., 2025). Mirabegron, a β3AR agonist, has been evaluated in clinical studies on heart failure. A 7-day course at 300 mg daily was sufficient to improve cardiac index in HFrEF patients, making mirabegron therapy promising (Bundgaard et al., 2022). In a rat model of PO-induced heart failure and in the ischemic human failing hearts, malic enzyme 1 (ME1) is upregulated, catalyzing anaplerosis that consumes NADPH and jeopardizes antioxidant capacity. ME1 suppression via miR-ME1 overexpression attenuated anaplerosis, replenished glutathione, and drove functional improvement in the PO-induced rat failing hearts (Lahey et al., 2018). Since all ME1 inhibitors remain in preclinical development, AAV9-based miR-ME1 overexpression represents a more feasible clinical strategy. In the mouse and human ischemic failing hearts, Epac1 expression is elevated, promoting CaMKII-mediated IDH2 inactivation. In preclinical models, NADPH levels can be restored using the CaMKII inhibitor KN93, which enhances cardiac antioxidant capacity (Fazal et al., 2017). For clinical studies, the CaMKII inhibitor CRD-4730 serves as a promising drug candidate for heart failure management. In the infarcted mouse and human hearts, NFE2L3 is upregulated, suppressing the expression of SOD2, GPX4, and peroxiredoxin 2. Genetic deletion of NFE2L3 in the mouse failing hearts normalized antioxidant gene expression, enhanced ROS scavenging capacity, and attenuated MI-induced contractile dysfunction and left ventricular (LV) dilation (Chen et al., 2025). Moreover, LARP7 expression is downregulated by ataxia telangiectasia mutated protein (ATM) in the DCM-affected human failing hearts and the infarcted mouse hearts, resulting in reduced expression of antioxidant genes. Pharmacological inhibition of ATM with KU60019 prevented LARP7 degradation, restored the expression of SIRT1, SOD2, and catalase, and attenuated deterioration of systolic function in the ischemic mouse failing hearts (Yu et al., 2021). Although several ATM inhibitors have entered human clinical trials, they are being developed exclusively as cancer therapies (Siu et al., 2025). Whether ATM inhibitors hold therapeutic promise for heart failure remains to be determined.

Antioxidant supplementation has also been extensively evaluated, with coenzyme Q_10_ and MitoQ demonstrating potent ROS-scavenging activity. Coenzyme Q_10_ is a component of the mitochondrial electron transport chain, where it transports electrons from Complex I or II to Complex III (Pallotti et al., 2021). Coenzyme Q_10_ deficiency has been identified in individuals with heart failure, leading to respiratory dysfunction and increased ROS production (Al Saadi et al., 2021). Coenzyme Q_10_ has exhibited cardioprotective effects in preclinical models of HFrEF, attenuating cardiac dysfunction and myocardial infarct size (Awad et al., 2022). In clinical settings, results from Q-SYMBIO (a randomized double-blind trial) and Long-term Outcome trial suggested that coenzyme Q_10_ treatment at 300 mg/day for 2 years significantly reduced cardiovascular mortality and incidence of hospital stays for heart failure (Mortensen et al., 2014). However, owing to its inability to passively traverse the mitochondrial membrane and adequately replenish endogenous levels, coenzyme Q_10_ requires large doses (2400-3000 mg/day) to produce beneficial effects in adults with heart failure (Ferrante et al., 2005; McGarry et al., 2017). Mitoquinone mesylate (MitoQ) is a coenzyme Q_10_ derivative engineered for mitochondrial targeting by conjugating the coenzyme Q_10_ moiety to a lipophilic cation, dTPP. By moving against its concentration gradient, dTPP accumulates within mitochondria, thereby improving the efficiency of coenzyme Q_10_ delivery to the organelle (Zielonka et al., 2017). MitoQ confers cardioprotective benefits in preclinical models of heart failure and clinical models of oxidative stress-associated pathologies (Kim et al., 2020; Rossman et al., 2018; Snow et al., 2010). However, a 12-month course of MitoQ at 40 mg/day failed to improve myocardial energetics or contractile function in DCM patients, suggesting that oxidative stress is not a central mediator of energetic impairment in DCM (Halliday et al., 2026). The identification of novel ROS-scavenging compounds provides a means to surmount translational hurdlers such as lack of tissue permeability and poor intracellular targeting. Treatment with MitoTEMPO, XJB-5-131, and EUK8/EUK134 improved cardiac recovery and contractile properties in preclinical HFrEF models and therefore holds promise for translational studies (Dikalova et al., 2010; Escobales et al., 2014; Koyama et al., 2013).

### 12.4 Therapeutic strategies targeting mitochondrial membrane permeability transition in HFrEF

Increased proclivity of mPTP opening have been observed in animal and human HFrEF models, contributing to apoptotic cell death and myocardial injury (Halestrap et al., 2004; Halestrap and Pasdois, 2009). Therapeutic strategies should therefore be directed at Ca^2+^ handling proteins and the mPTP regulatory machinery to alleviate the associated damage.

Therapeutic strategies targeting mitochondrial Ca^2+^ have proven effective in restraining mPTP opening and mitigating cell death. Increased Epac1 expression in the human (with ICM or DCM) and mouse (with ICM) failing hearts enhances SR-mitochondria Ca^2+^ transfer, leading to mitochondrial Ca^2+^ overload and triggering mPTP opening. Mitochondrial membrane permeability transition can be suppressed by the selective Epac1 inhibitor CE3F4, which blocks mPTP opening, mitigates cardiomyocyte apoptosis, and reduces infarct size in the mouse failing hearts (Fazal et al., 2017). However, an Epac1-selective inhibitor for clinical trials in heart failure is currently unavailable. GRK2, which increases the sensitivity of mPTP to Ca^2+^, is upregulated in the human (with DCM) failing hearts and in a mouse model of ischemia- or PO-induced heart failure. In preclinical studies, treatment of cardiomyocytes with the mitogen-activated protein kinase kinase (MEK) inhibitor PD98059 prevented ERK-mediated GRK2 phosphorylation and mitochondrial translocation, and suppressed stress-induced cell death (Chen et al., 2013). Clinical trials utilizing the GRK2 inhibitor paroxetine reduced infarct size in MI patients after 12 weeks of treatment but did not lead to functional improvement at 1 year, possibly attributable to the low paroxetine dose (20 mg/day) (Roy and Koch, 2026). However, dose escalation must be approached with caution, given that higher doses are associated with stronger off-target effects.

Therapies directed at CypD, the positive regulator of mPTP, also exhibit cardioprotective effects. The expression of mecciRNAs, which maintain high TRAP1 and low CypD levels in mitochondria, is downregulated in the DCM-affected human failing hearts and the PO-induced mouse failing hearts, leading to an enhanced propensity for mPTP opening. Administration of mecciND2 in the mouse failing hearts inhibited mPTP opening, delayed the transition to decompensation, and conferred protection against heart failure (Liu et al., 2025). However, therapies involving mecciRNA delivery has not been developed in clinical models, despite an urgent need. HR enhances SR-to-mitochondria Ca^2+^ transfer and triggers mPTP opening by consolidating the CypD/VDAC1/Grp75/IP3R1 complex. In preclinical studies, interactions among the CypD/VDAC1/Grp75/IP3R1 complex members were disrupted upon treatment with a specific CypD inhibitor, NIM811, which reduced mitochondrial Ca^2+^ accumulation and inhibited HR-induced mPTP opening and cell death (Paillard et al., 2013). Despite these preclinical benefits, NIM811 has not entered clinical trials for heart failure therapy. HO-1 inhibits mPTP opening in a CO-dependent manner and is upregulated in the human (with DCM or ICM) and mouse (with ICM) failing hearts. Both cardiac-specific HO-1 overexpression and treatment with the CO donor CORM-3 inhibited mitochondrial membrane permeability transition and attenuated LV dilatation and systolic dysfunction in the infarcted mouse hearts (Wang et al., 2010). However, research into CO-releasing molecules (CORMs) remains entirely preclinical, with no human trials registered for heart failure. Pim-1 is another mPTP inhibitor, and its mitochondrial translocation is enhanced in the mouse hearts in response to I/R injury. Preclinical studies showed that cardiomyocytes overexpressing Pim-1 exhibited resistance to ROS-induced mitochondrial inner membrane depolization, which was associated with reduced mPTP opening and apoptosis (Borillo et al., 2010). The success of Pim-1 overexpression as a preclinical therapy may pave the way for further clinical studies using AAV9 as the gene delivery method.

Cyclosporin is a potent inhibitor of mPTP opening that operates through its binding to CypD. In a clinical trial for cyclosporin, a daily dose of 2.5 mg/kg body weight induced a persistent reduction in infarct size at 6 months, accompanied by attenuated LV dilation and improved LV ejection fraction (Mewton et al., 2010; Piot et al., 2008). However, chronic cyclosporin administration has been linked to immunosuppression and renal pathology (Naesens et al., 2009; Tábara et al., 2014). Therefore, cyclosporin treatment is not an appropriate approach for long-term heart failure management.

### 12.5 Therapeutic strategies targeting mitophagy in HFrEF

Mitophagy, a selective form of autophagy that degrades dysfunctional mitochondria, is impaired during heart failure. Therapeutic strategies aimed at restoring mitochondrial quality control are directed at the machinery and pathways regulating ubiquitin- or receptor-mediated mitophagy. Mitophagy is downregulated in both the human and mouse failing hearts and can be potentiated using the Tat-Beclin 1 peptide (TB1), which is a potent inducer of autophagy. TB1 treatment restored mitophagic flux, preserved LV ejection fraction, and attenuated cardiomyocyte apoptosis in the PO-induced mouse failing hearts (Shirakabe et al., 2016). However, TB1 is not a appropriate drug for clinical use owing to unintended, tissue-specific side effects (Rockel et al., 2020). The expression and activity of AMPKα2 are downregulated in the PO-induced mouse failing hearts and in human DCM and ischemic heart failure, suppressing PINK1/Parkin-dependent mitophagy. Cardiac-specific AMPKα2 overexpression enhanced the mitochondrial translocation of PINK1 and Parkin and potentiated their activities, mitigating adverse remodeling in the mouse model of PO-induced heart failure (Wang et al., 2018). Dapagliflozin, a sodium-glucose cotransporter-2 inhibitor, attenuates cardiac hypertrophy through activation of AMPKα2-dependent mitophagy (Feng et al., 2025). In a multicenter clinical trial, dapagliflozin at 10 mg/day for 16 weeks improved symptoms in patients with HFrEF (McMurray et al., 2024). The DAPA-HF trial showed that dapagliflozin at 10 mg/day for 18 months reduced the risk of worsening heart failure and death from cardiovascular causes (McMurray et al., 2019). Therefore, targeting AMPKα2-dependent activation of mitophagy is clinically plausible and has demonstrated effectiveness. The expression of SPOP, which promotes the ubiquitination and degradation of TFEB, is upregulated in human DCM and the mouse (with ICM or PO-induced) failing hearts. Treatment with the SPOP inhibitor I-6lc attenuated TFEB degradation, relieved the suppression of autophagy-related genes, and improved cardiac function in the PO-induced mouse failing hearts (Wu et al., 2025). No SPOP inhibitor has entered clinical trials for heart failure, and the feasibility of their clinical use requires further investigation. ANXA2 mediates the degradation of the mitophagy receptor PHB2 and is upregulated in both the human and mouse ischemic failing hearts. Cardiomyocyte-specific ANXA2 deletion restored PHB2 protein levels, enhanced mitophagic activity, and alleviated LV dilatation and contractile dysfunction in MI mice (Deng et al., 2026). However, an ANXA2 inhibitor is not currently available for clinical studies focused on heart failure.

## 13 Conclusions and future directions

Accumulating evidence has linked mitochondrial remodeling and dysfunction to the pathophysiology of heart failure, and considerable experimental and clinical efforts have been dedicated to improving heart failure outcomes utilizing mitochondria-targeted therapies. A central challenge is the difficulty of translating mechanistic insights into effective clinical interventions. Serving as the energy production center, mitochondria are intrinsically connected to nearly all aspects of cellular processes, rendering them a pivotal yet challenging therapeutic target. Their dynamic structure, double-membrane barrier, and interplay with cellular metabolism pose substantial obstacles to drug delivery, therapeutic precision, and efficacy.

Among the challenges facing mitochondria-targeted heart failure therapies is the need to attenuate off-target effects. These off-target effects can be grouped into several distinct categories, extending from unintended interference with physiological signaling to organ-specific adverse events. Cyclosporin’s therapeutic potential for chronic heart failure is fundamentally limited by its functional mechanism: as a CypD inhibitor, it also inhibits calcineurin and blocks T cell activation, producing severe off-target effects including immunosuppression and renal pathology (Rusnak and Mertz, 2000). Additionally, indiscriminate ROS scavenging by mitochondria-targeted antioxidants can disrupt physiological ROS signaling needed for preconditioning and mitochondrial quality control. In cells, MitoQ has been demonstrated to abolish H_2_O_2_-induced transactivation of the epidermal growth factor receptor, along with the downstream Akt and JNK signaling (Chen et al., 2004). The triphenylphosphonium (TPP) conjugation strategy employed by mitochondria-targeted compounds such as MitoQ exploits mitochondrial membrane potential as the driving force for accumulation. However, injured mitochondria frequently exhibit decreased mitochondrial membrane potential, leading to reduced drug uptake relative to healthy mitochondria; this may disrupt normal mitochondrial function while failing to adequately treat the damaged population. Enhancing delivery precision through nanocarriers provides a promising strategy to resolve the selectivity challenge. Nanocarriers, including liposomes and polymeric or inorganic nanoparticles, enable more direct drug delivery to mitochondria, thereby reducing off-target effects and enhancing mitochondrial targeting (Giannitti et al., 2025). Poly lactic-co-glycolic acid (PLGA) nanoparticles incorporating cyclosporin have shown promising results, exhibiting greater potency than free cyclosporin while lacking its immunosuppressive activity (Ikeda et al., 2016).

Another crucial challenge related to clinical translation is the development of reliable, validated probes for monitoring mitochondrial function in heart diseases. Current assessments predominantly rely on indirect markers, including oxidative stress indicators and circulating lactate, all of which suffer from either nonspecificity or high invasiveness (Paraskevaidis et al., 2024). Circulating mtDNA and oxidized cardiolipin have emerged as alternative markers for monitoring mitochondrial health, given their correlation with inflammation, mitochondrial membrane destabilization, and other adverse outcomes in cardiovascular diseases (Iyer et al., 2013; Zhang et al., 2010). Persistent shortcomings inherent to preexisting probes complicate efforts to develop strategies with therapeutic precision. Measuring the carbonylation of cardiac proteins has been used to monitor oxidative stress in heart failure, but the strategy fails to identify the ROS source (Aryal et al., 2014). MitoTimer permits fluorescence microscopy-based assessment of mitochondrial morphology and redox state, but only in fixed tissues (Stotland and Gottlieb, 2016). Real-time monitoring of mitochondrial dynamics over the course of heart failure progression is required, given that current studies predominantly rely on single time-point analyses. *In vivo* dynamic nuclear polarization MRI (DNP-MRI), paired with the nitroxyl probe carbamoyl-PROXYL (CmP), permits noninvasive visualization of ROS in living cardiomyocytes (Ichihashi et al., 2026). This approach enables detection of redox imbalance prior to the emergence of any stress-induced functional or morphological changes. Furthermore, combined imaging-therapy platforms, where a single probe simultaneously visualizes mitochondrial dysfunction and delivers therapeutic benefit, constitute a particularly promising direction, as exemplified by IR-780 and the ESC-AGO-2 system (Chen et al., 2023; Du et al., 2025).

The heterogeneity of mitochondrial remodeling across different heart failure etiologies demands therapeutic strategies with etiology-specific and phenotype-specific targeting. Because mitochondrial abnormalities differ across heart failure contexts, treatment strategies should be tailored rather than one-size-fits-all (Yang et al., 2026). Individualized treatment regimens should be guided by specific mitochondrial signatures derived from genomic, proteomic, and metabolomic profiling (Murphy and Hartley, 2018). Moreover, the interconnected nature of mitochondrial functions determines that remodeling in one process propagates a pathological signaling cascade that drives mitochondrial dysfunction across multiple related processes. This provides a clear rationale for combination strategies that concomitantly address multiple nodes of mitochondrial dysfunction, rather than single-agent approaches, in light of the multifaceted nature of mitochondrial pathology.

In summary, mitochondria-targeted therapies for heart failure represent a field of immense promise but substantial challenge. Future efforts should prioritize several interconnected goals: the attenuation of unintended off-target effects to improve therapeutic precision, the development of etiology-specific and phenotype-driven therapeutic approaches, the integration of combination strategies that address multiple nodes of mitochondrial dysfunction, and the validation of reliable biomarkers and imaging probes to monitor target engagement and therapeutic response. The successful translation of mechanistic insights into adequately powered therapeutic strategies will open new avenues for cardioprotection, enhance functional capacity, and improve survival for millions of patients affected by heart failure worldwide.
